# Characterizing Aptamers with Reconfigurable Chiral Plasmonic Assemblies

**DOI:** 10.1021/acs.langmuir.1c03434

**Published:** 2022-02-25

**Authors:** Yike Huang, Minh-Kha Nguyen, Vu Hoang Nguyen, Jacky Loo, Arttu J. Lehtonen, Anton Kuzyk

**Affiliations:** † Department of Neuroscience and Biomedical Engineering, School of Science, Aalto University, FI-00076 Aalto, Finland; ‡ Faculty of Chemical Engineering, Ho Chi Minh City University of Technology (HCMUT), 268 Ly Thuong Kiet Street, Dist. 10, 700000 Ho Chi Minh City, Vietnam; § Vietnam National University Ho Chi Minh City, Linh Trung Ward, Thu Duc District, 700000 Ho Chi Minh City, Vietnam; ∥ Department of Electrical Engineering and Automation, School of Electrical Engineering, Aalto University, FI-00076 Aalto, Finland

## Abstract

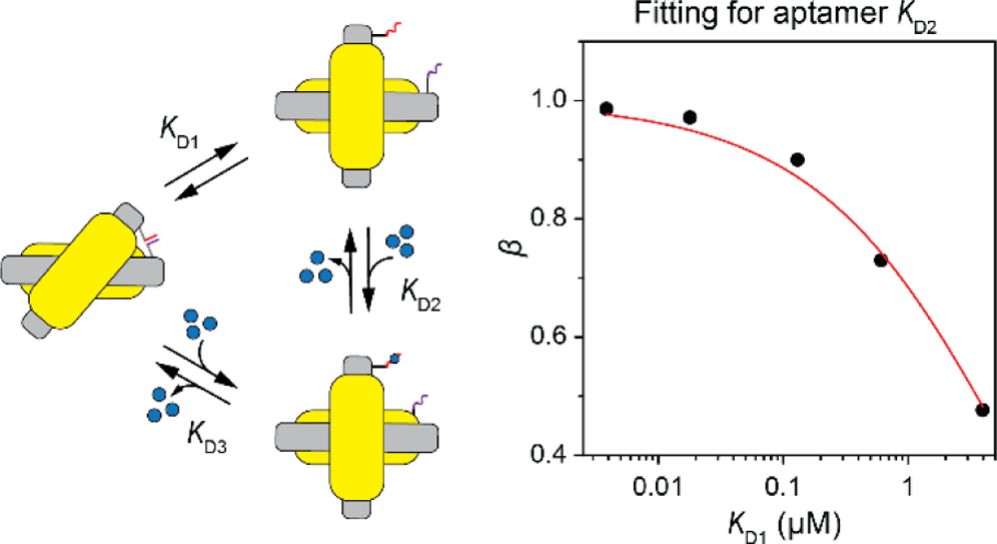

Aptamers have emerged as versatile affinity ligands and as promising alternatives to protein antibodies. However, the inconsistency in the reported affinities and specificities of aptamers has greatly hindered the development of aptamer-based applications. Herein, we present a strategy to characterize aptamers by using DNA origami-based chiral plasmonic assemblies as reporters and establishing a competitive hybridization reaction-based thermodynamic model. We demonstrate the characterization of several DNA aptamers, including aptamers for small molecules and macromolecules, as well as aptamers with high and low affinities. The presented characterization scheme can be readily adapted to a wide selection of aptamers. We anticipate that our approach will advance the development of aptamer-based applications by enabling reliable and reproducible characterization of aptamers.

## Introduction

Nucleic acid aptamers, that is, single-stranded DNA or RNA oligonucleotides that selectively and specifically bind to targets of interest with high affinity, are promising affinity ligands with a wide range of potential applications in biosensing, therapeutics, and synthetic chemistry.^1−5^ Despite the merits, inconsistency in aptamers’ characterizations has hindered the advancement of aptamer-based applications.^6,7^ Although over 1000 aptamers have been reported in the literature, rather limited sets of ∼15 well-characterized aptamer–target pairs have been used in the majority of research articles dealing with application development.^2^ Currently, the characterization of the aptamers’ affinities typically relies on (i) measurement of the enthalpy changes (e.g., using isothermal titration calorimetry),^8,9^ which usually requires a significant amount of samples, (ii) measurement of the association and dissociation rates (e.g., using surface plasmon resonance),^10^ which often needs immobilization or labeling that might disturb the aptamer–analyte interactions,^11−13^ or (iii) measurement of concentration-dependent fractions of bound and unbound aptamers at equilibrium using a wide range of available techniques.^14^ However, partitioning the bound and unbound aptamers often relies on specific characteristics of a particular analyte–aptamer complex (size, charge, and structural change, etc.), and the choice of characterization method might not be optimal due to the specific lab resources.^15^ A general and reliable approach to quantify the binding affinity and specificity of aptamers targeting different molecules is needed.^16−19^ Such a general method should be suitable for the characterization of aptamers targeting molecules of different sizes (from small molecules to proteins) with a broad range of affinities [equilibrium dissociation constant (*K*_D_) values spanning nM to mM range]. Taking advantage of the DNA origami-based reconfigurable chiral plasmonic assemblies,^20,21^ here we propose a competitive hybridization reaction-based strategy, which can be applied for characterizing a wide range of aptamer–analyte pairs. Compared to the traditional hybridization reaction-based methods where a complementary strand is fixed and the concentration dependency is used to quantify the binding affinity,^22^ our approach varies the complementary strands both in the hybridization regions and lengths at a fixed analyte concentration. We tested our approach by characterizing ATP, thrombin, and glucose DNA aptamers, which constitute small molecule—intermediate *K*_D_ (μM range), protein—low *K*_D_ (nM range), and small molecule—high *K*_D_ (mM range) cases, respectively.

## Results and Discussion

**Figure 1:**
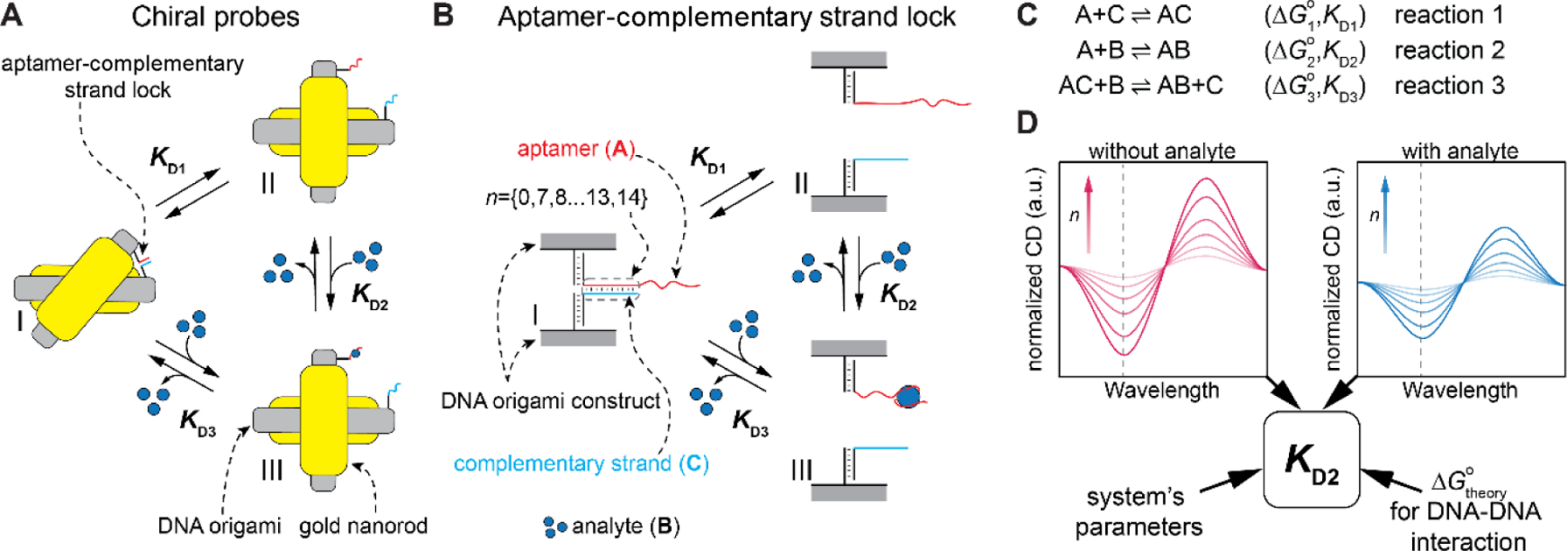
(A) Schematics of DNA origami-based chiral plasmonic probes. The aptamer and a partially complementary strand are incorporated into the probe as an analyte responsive lock. The hybridized state of the two strands corresponds to the closed configuration of the probe (I); the separation of the strands results in open probe configurations (II, III). (B) Configurations of aptamer locks with varied lengths of hybridization (*n*) between the aptamer and the complementary strand. The hybridization length of *n* = 0 bp corresponds to the open lock. Increasing *n* shifts the equilibrium toward the hybridized state of the lock and the closed configuration of the chiral probes. (C) Description of the system in terms of chemical reactions: A, B, and C are the aptamer, analyte, and complementary strand, respectively; Δ*G*° and *K*_D_ are the corresponding Gibbs free energy and dissociation constants, respectively, with *K*_D2_ being the aptamer–analyte dissociation constant of interest. (D) Brief overview of data analysis for the determination of *K*_D2_. The probes with the aptamer and partially complementary strands of different lengths are incubated with or without the analyte. With the parameters obtained from the calibration experiments and computational prediction tools, *K*_D2_ is calculated by measuring the amplitude changes of the CD spectra in the presence and absence of the analyte.

Our approach to aptamer characterization is schematically illustrated in Figure 1. Reconfigurable chiral plasmonic probes comprising two gold nanorods (AuNRs) are fabricated using DNA origami-guided assembly^23−33^ (Figure 1A; for design and fabrication details, see Section S1). An aptamer (A) and a partially complementary strand (C) are incorporated into a probe as an analyte responsive “lock” (Figures 1B and S2). The hybridized state of A and C strands corresponds to the closed right-handed chiral configuration of the probe with a strong circular dichroism (CD) response. The separation of A and C strands, that is, opening the lock, results in the open probe configuration with a weak CD response originating from the residual chirality.^27^ In the presence of an analyte (B), the dynamic equilibrium of the probe ensemble is described by three different states (Figure 1A,B): (1) closed configuration I with A and C strands being hybridized; (2) open configuration II with A (without bound analyte) and C being separated; (3) open configuration III with A (with bound analyte) and C being separated. In terms of chemical equilibrium, the system can be described by three reactions (Figure 1C). The hybridization of the A and C strands (reaction 1) and the aptamer–analyte binding (reaction 2) compete against each other.

The equilibrium concentration of AC hybrid, corresponding to the concentration of the probe in the closed configuration I, is different in the presence and absence of the analyte resulting in different CD responses (Figure 1D). To obtain the aptamer–analyte dissociation constant (*K*_D2_) from the measured optical responses, we introduced parameter β as the ratio of AC hybrid concentrations in the presence and absence of the analyte (eq S9). As deduced in the Supporting Information (Section S2.1), on the one hand, β is a function of the input local concentration of A and C strands (*a*_0_), the bulk concentration of the analyte (*b*_0_), and the dissociation constants of hybridization (*K*_D1_) as well as *K*_D2_, that is, β = *f*(*a*_0_, *b*_0_, *K*_D1_, *K*_D2_) (eq S9); on the other hand, β equals to the ratio of the relative normalized CD amplitudes in the presence and absence of the analyte (eq S26) and can be obtained from the experimental measurements. As *b*_0_ is a known constant in the experiment, to obtain *K*_D2_, values of *K*_D1_ and *a*_0_ must be determined first.

**Figure 2:**
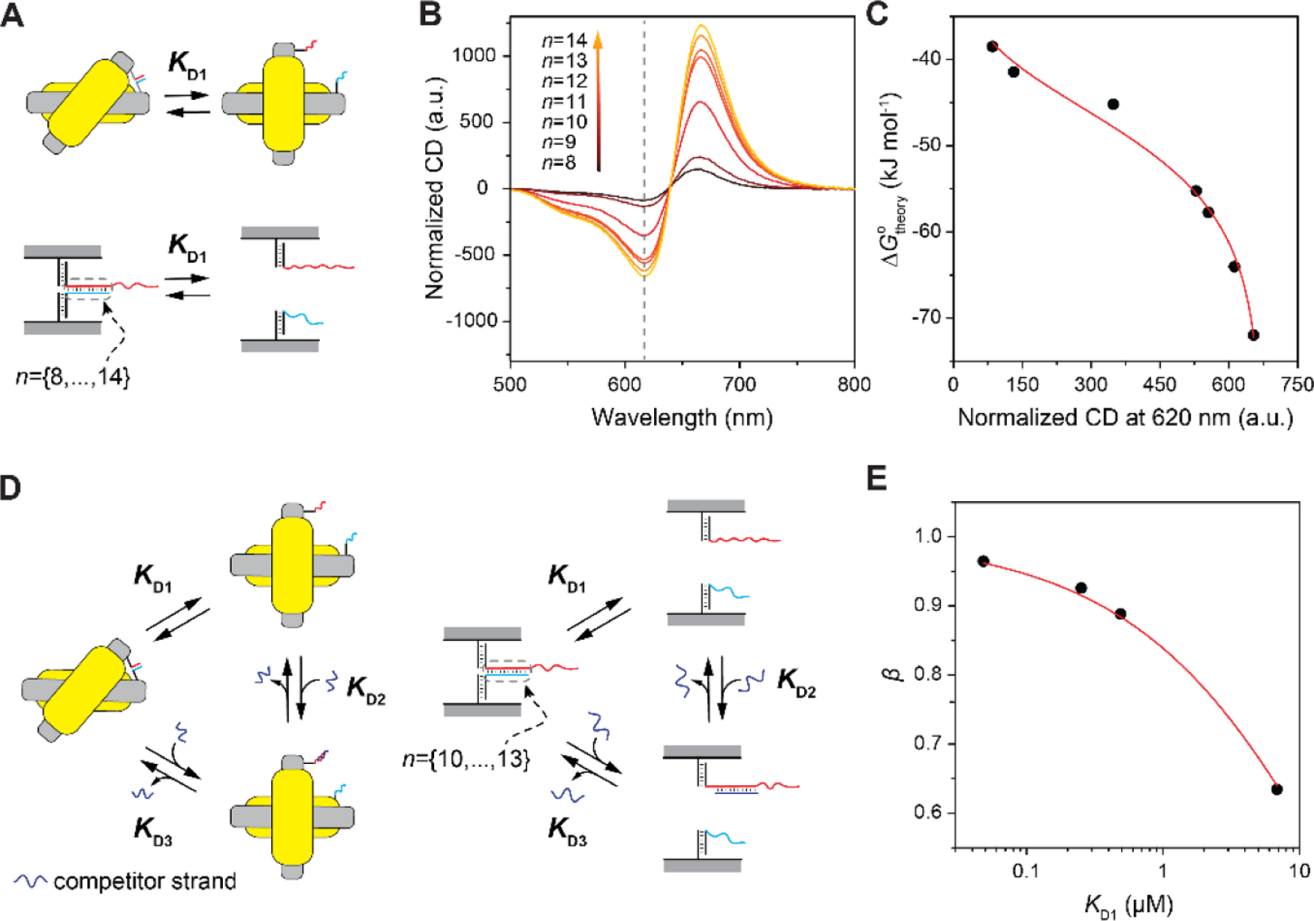
(A) To obtain the coefficient ε between the predicted (Δ*G*_theory_^°^) and real (Δ*G*_r_^°^) Gibbs free energies, the hybridization length (*n*) between the aptamer and the complementary strand is varied from 8 to 14 bp (*n* = {8–14}). (B) Normalized CD spectra of the probes with different hybridization lengths (*n*). (C) Dependence of Δ*G*_theory_^°^(*n*) on the normalized CD signal at 620 nm is used to calculate ε. (D) To obtain the local concentration of A and C strands (*a*_0_), *n* is varied between 10 and 13 bp (*n* = {10–13}). A competitor DNA strand (10 nt) is used as an analyte. (E) By varying *n*, the dependence of β on *K*_D1_ is used to calculate *a*_0_.

In principle, *K*_D1_ can be directly calculated from the Gibbs free energy for hybridization between A and C strands (reaction 1, Figure 1C) as  using Δ*G*_theory_^°^ values provided by computational tools, for example, mfold^34^ or NUPACK.^35^ However, the real Gibbs free energy (Δ*G*_r_^°^) in a specific nucleic acid system is often different from Δ*G*_theory_^°^ due to the change of enthalpy and entropy in a particular microenvironment.^36,37^ To compensate for this discrepancy, we introduced a coefficient ε = Δ*G*_r_^°^/Δ*G*_theory_^°^ (see also Section S2.2 and Figure S3). To obtain the value of ε, we used a template strand with a fixed sequence as A and a set of its partially complementary strands as C. We varied the AC hybridization length (*n*) between *n* = 8 and *n* = 14 base pairs (bp) and measured the CD signals (Figure 2A). A single base pair difference in the hybridization of the template strand-complementary strand resulted in clearly different CD responses (Figure 2B). The dependence of calculated Δ*G*_theory_^°^ on the normalized measured CD was fitted using eq S34, and the fitting resulted in a ε value of 0.65 (Figure 2C).

Next, we evaluated *a*_0_. It is important to note here that the interaction between A and C strands depends on the local rather than the global concentrations as interacting strands are linked to the same DNA origami construct. To obtain *a*_0_, we used a 10 nucleotide (nt) competitor strand (S) and varied the hybridization lengths between A and C strands from *n* = 10 to 13 bp (Figure 2D, see also Section S2.2). The presence of S shifts the equilibrium toward the open configuration and decreases the CD signals (Figure S10). The real Gibbs free energies (Δ*G*_r_^°^) of the A + C(*n*) ⇌ AC(*n*) and A + S ⇌ AS reactions were obtained from the theoretical Gibbs free energies (Δ*G*_theory_^°^) predicted by mfold and corrected with ε. The corresponding *K*_D1_(*n*) and *K*_D2_^S^ values were calculated with . The CD spectra generated by the probes with different *n* were measured in the presence and absence of S, and the corresponding ratios (β_*n*_) of the relative normalized CD were gained. By fitting the dependence of β_*n*_ on *K*_D1_(*n*) using eq S38 (Figure 2E), we obtained the *a*_0_ value of 76 ± 3.8 μM. Note that ε and *a*_0_ are system parameters which depend on the design of the chiral probes but not on the sequences of the lock strands. Once determined, the values of ε and *a*_0_ can be used for aptamer affinity (*K*_D2_) calculation as long as the design of the chiral probes is not altered.

**Figure 3:**
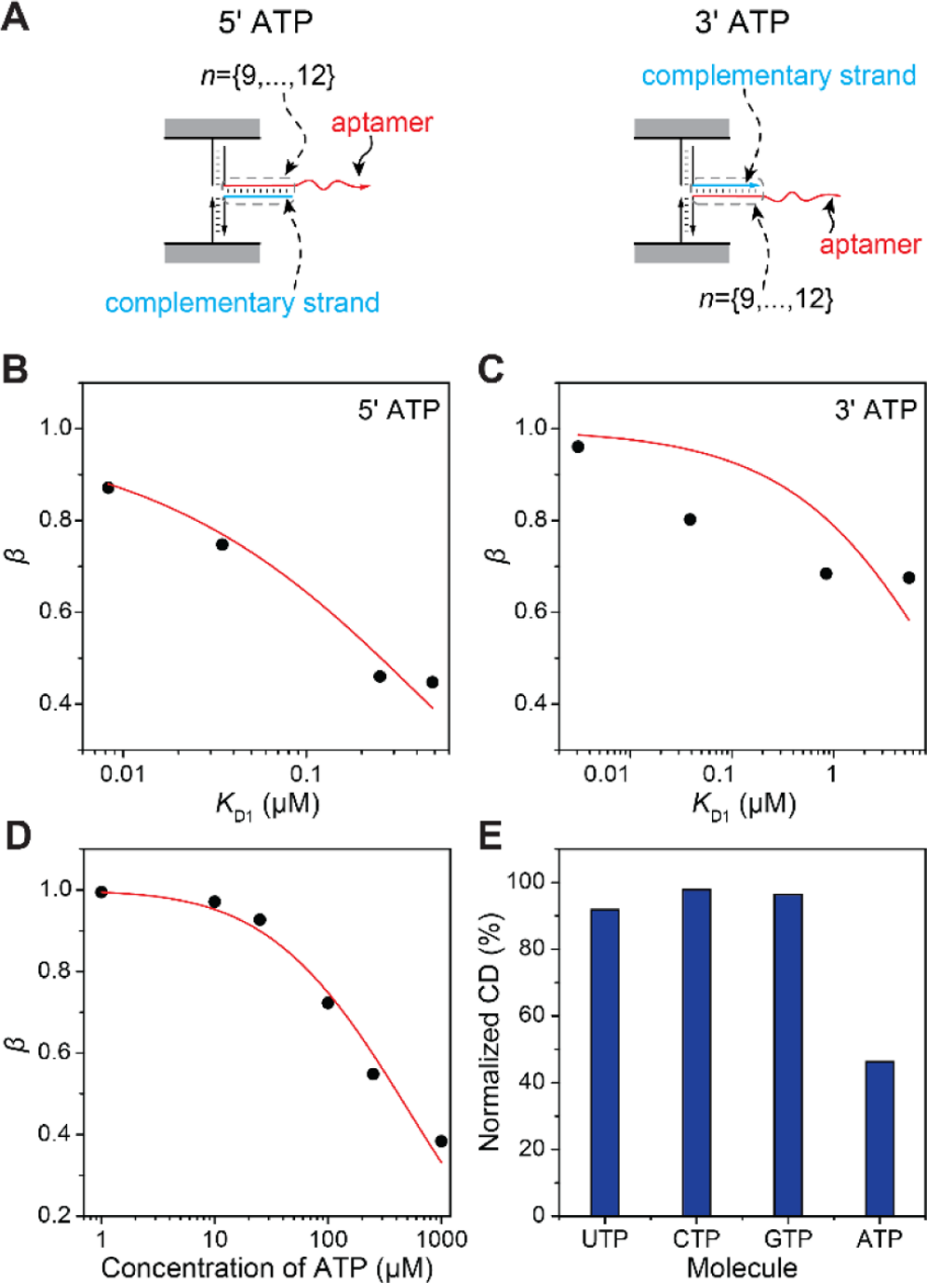
(A) Hybridization of complementary strands to the 5′ or 3′ end of the ATP aptamer with hybridization lengths varied between 9 and 12 bp (*n* = {9–12}). (B,C) Dependence of β_*n*_ on *K*_D1_(*n*) with the complementary strand hybridizing at the 5′ (B) or 3′ (C) end of the ATP aptamer. *K*_D2_^ATP^ values of 5.71 ± 0.865 μM (B) and 117 ± 62.8 μM (C) were obtained from the fitting. (D) Dependence of β on the ATP concentration (*b*_0_) produced *K*_D2_^ATP^ values of 4.54 ± 0.580 μM. (E) Specificity characterization of the ATP aptamer (see Figure S12 for CD spectra).

To test our approach to aptamer characterization (for complete workflow, see Figure S4), we first investigated the well-studied ATP DNA aptamer.^38^ We inserted the ATP aptamer (as A strand) and a set of its partially complementary strands (as C strands) into the chiral probes. The hybridization lengths between A and C strands were varied between *n* = 9 and 12 bp either on the 5′ or 3′ end of the aptamer (Figure 3A). For each pair of A and C strands, *K*_D1_(*n*) was calculated using Δ*G*_theory_^°^ and ε, and β_*n*_ was obtained from the CD measurements (Figure S12). The input concentration of ATP was fixed at 1 mM. For the set of C strands hybridizing to the 5′ region of the ATP aptamer, *K*_D2_^ATP^ of 5.71 ± 0.865 μM was obtained from fitting the β_*n*_ dependence on *K*_D1_(*n*) using eq S38 (Figure 3B, fitting coefficient of determination *R*^2^ = 0.96). The obtained *K*_D2_^ATP^ value agreed well with previous reports.^10,38−40^ For the set of C strands hybridizing to the 3′ region of the ATP aptamer, the *K*_D2_^ATP^ value of 117 ± 62.8 μM was gained (Figure 3C, *R*^2^ = 0.13).

The discrepancy between the 5′ and 3′ hybridizations possibly originates from a kinetic trap or a side product of the reaction.^41^ Due to the nonhomogeneous nature of the aptamer sequence, different domains of the aptamer interact with the analyte in various kinetic and thermodynamic behaviors. Typically, the whole aptamer sequence can be divided into three domains: (i) nonessential sequence which neither interacts with the analyte nor supports structural folding and should be truncated, (ii) essential but noncritical sequence which is not critical for the initial interaction of the analyte but essential for analyte binding, and (iii) critical sequence plays an important role in the initial interaction of the analyte. The calculated *K*_D2_ is not valid if the hybridization between A and C strands hinders the system from reaching the equilibrium, for example, when the critical domain of the aptamer is blocked and AC dissociation rate is too slow. Also, the correct *K*_D2_ can be obtained only when the A and C strands are fully dissociated upon analyte binding, avoiding the three-molecule (analyte–aptamer-complementary strands) complex formation caused by hybridization to the nonessential domain. Hence, to exclude the wrong choice of the complementary domain, we use the goodness of the fitting (*R*^2^) to judge the validity of obtained *K*_D2_ values and set the validity threshold at *R*^2^ = 0.95 (see detailed discussion in Section S3). Therefore, *K*_D2_^ATP^ of 5.71 μM with *R*^2^ = 0.96, which was obtained using the set of complementary strands that hybridize at the 5′ end of the aptamer, can be considered as a valid value. The goodness of the fitting was only 0.13 with the aptamer-complementary strands hybridizing at 3′, indicating a poor validity of the obtained dissociation constant due to the wrong choice of the blocking domain.

To further confirm the characterization results of the ATP aptamers, we varied the concentration of ATP from 1 μM to 1 mM to obtain *K*_D2_^ATP^ using the dependence of β on the analyte concentration, that is, *b*_0_ (see eq S38). With the thermodynamic and kinetic information gained from varying the hybridization lengths and regions, we fabricated chiral probes with the complementary strands forming 9 base pairs to the 5′ end of the ATP aptamer. From the β dependence on *b*_0_, we obtained a *K*_D2_^ATP^ value of 4.54 ± 0.580 μM (Figure 3D). We used the same chiral probes (*n* = 9 at 5′ end) to evaluate the specificity of the ATP aptamer. After incubation in 1 mM UTP, CTP, GTP, and ATP, the relative changes of the normalized CD signals were 8.1, 2.1, 3.6, and 53.7%, respectively, confirming the aptamer specificity toward ATP, relative to UTP, CTP, and GTP (Figures 3E and S12).

**Figure 4:**
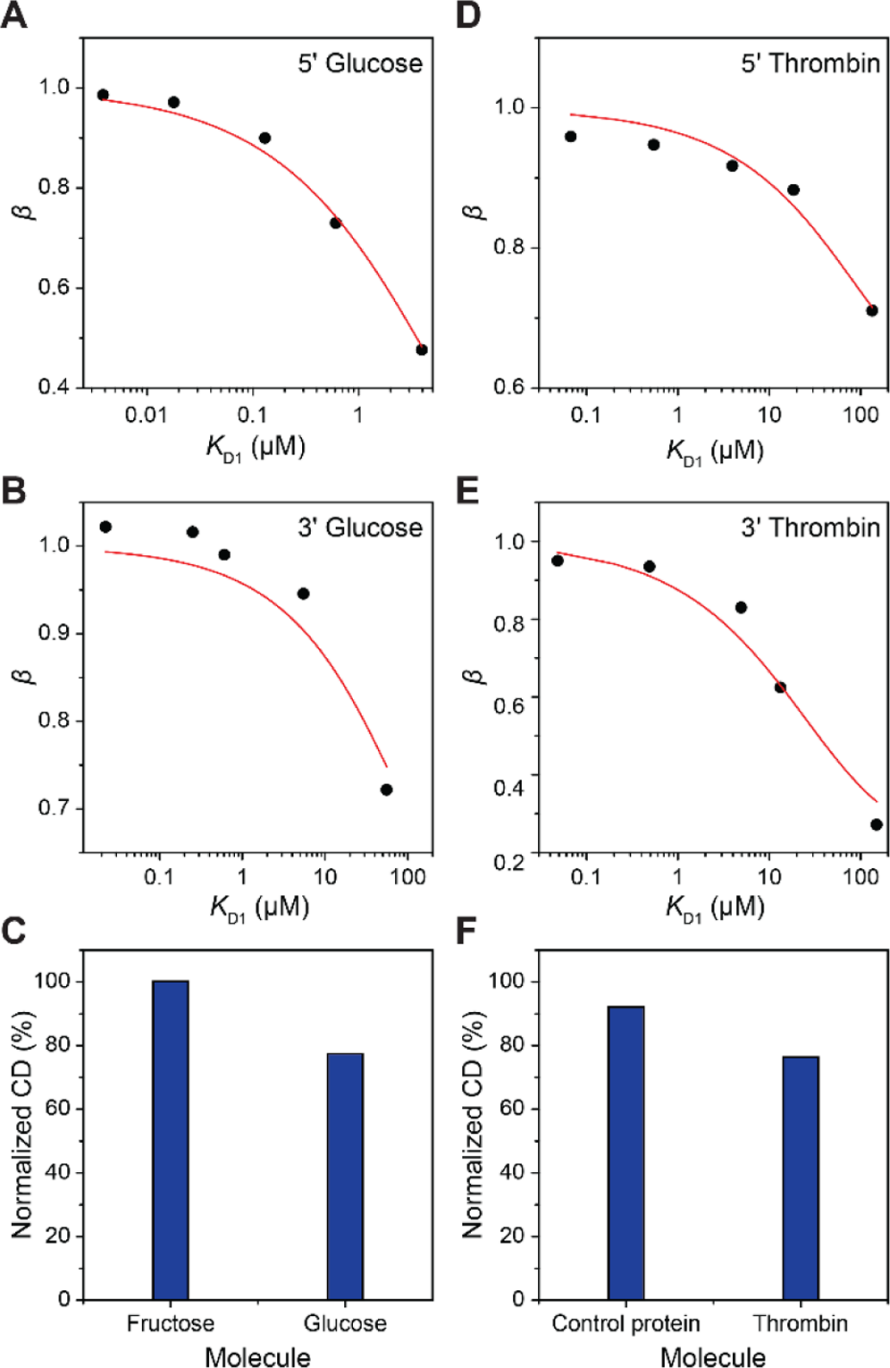
(A,B) Dependence of β_*n*_ on *K*_D1_(*n*) with complementary strand hybridizing at the 5′ (A) or 3′ (B) of the glucose aptamer.^42^*K*_D2_^glu^ values of 5.57 ± 0.436 mM (A) and 110 ± 20.0 mM (B) were obtained from the fitting. (C) Specificity characterization of the glucose aptamer (see Figure S13 for CD spectra). (D,E) Dependence of β_*n*_ on *K*_D1_(*n*) with the complementary strand hybridizing at 5′ (D) or 3′ (E) of the thrombin aptamer.^43^*K*_D2_^thr^ values of 235 ± 24.1 nM (D) and 46.7 ± 7.27 nM (E) were obtained from the fitting. (F) Specificity characterization of the thrombin aptamer (see Figure S14 for CD spectra).

Finally, we used our approach to evaluate *K*_D_ values of two additional aptamers. The recently selected glucose aptamer^42,44^ with a dissociation constant around 10 mM was used to demonstrate the validity of our approach for the characterization of aptamers with low affinity. The chiral probes with the hybridization lengths between A and C strands varying from *n* = 8 to *n* = 12 bp were incubated with 100 mM glucose (see Figure S13 for CD measurements). From the dependence of β_*n*_ on *K*_D1_(*n*), the *K*_D2_^glu^ values of 5.57 ± 0.436 mM (*R*^2^ = 0.99) and 110 ± 20.0 mM (*R*^2^ = 0.92) were obtained for the aptamer-complementary strand hybridization on the 5′ and 3′ ends, respectively (Figure 4A,B). We consider *K*_D2_^glu^ of 5.57 mM to be the valid value as *R*^2^ > 0.95 for the fitting. To evaluate the specificity of the glucose aptamer, chiral probes with A and C hybridization length of *n* = 10 bp at the 5′ end were incubated with 100 mM fructose and glucose. The relative normalized CD signal showed no change in the presence of fructose, while 22.6% decrease was observed with glucose, hence confirming the glucose aptamer specificity (Figures 4C and S13). The chiral probes containing locks without the glucose aptamer sequence did not exhibit change in CD response after incubation with glucose (Figure S15).

To evaluate the applicability of our approach for the characterization of protein aptamers, we incorporated a thrombin aptamer^43^ and the corresponding complementary strands in the chiral probes (*n* varied from 7 to 11 bp at the 5′ or 3′ end region of the aptamer). The probes were incubated with 170 nM thrombin, and *K*_D2_^thr^ values of 46.7 ± 7.27 nM (*R*^2^ = 0.97) and 235 ± 24.1 nM (*R*^2^ = 0.94) were obtained from the fitting of β_*n*_ on *K*_D1_(*n*) with the hybridization regions at the 3′ and 5′ ends of the aptamer, respectively (Figure 4D,E, see Figure S14 for CD measurements). Our results are in good agreement with previous publications^43,45^ that reported thrombin aptamer dissociation constants in the range of 25–200 nM. The specificity of the thrombin aptamer was evaluated with the chiral probes with the lock of *n* = 8 bp at the 3′ end. The probes were incubated with thrombin at 170 nM and the protein ladder containing protein molecules of different sizes (total protein concentration ∼ 2 μM). The relative normalized CD signal decreased by 7.9% and 23.6% after incubation with the protein ladder and thrombin, respectively (Figures 4F and S14). The chiral probes containing locks without the thrombin aptamer sequence did not exhibit change in CD response after incubation with thrombin (Figure S15).

## Conclusions

We developed a method for characterizing aptamers’ affinity and specificity using DNA origami-based reconfigurable chiral plasmonic assemblies. Our method is applicable to a wide range of aptamers targeting molecules of different sizes with a broad range of affinities. Neither the aptamers nor the analyte requires modification/labeling or surface immobilization, so the method enables aptamer characterization close to the native state. Furthermore, the CD-based approach allows optical characterization in nontransparent environments, hence relaxing the requirements for sample preparation and purification.

Compared to traditional methods, where the concentration of the analyte is varied, our approach varies the hybridization between an aptamer and its partially complementary strands. This enables obtaining important insights on the hetero domains of the aptamer sequence. By varying the complementary strands and using the goodness of fitting (*R*^2^), the risk of choosing the hybridization that causes the formation of kinetic traps and/or side products is significantly reduced and the validity of the *K*_D_ measurements is ensured.

Our approach, in principle, can be readily extended to mapping over the whole aptamer sequence, with goodness of fitting providing valuable information on the reliability of the obtained *K*_D_ values. Our results demonstrate a promising route toward the development of bioaffinity characterization platforms utilizing optical responses of reconfigurable chiral plasmonic assemblies.

## Materials and Methods

### Fabrication of DNA Origami Structures with Aptamer and Complementary Strands

Aptamer (template strand) and its complementary strands together with staple strands were mixed with DNA scaffold p7560 (purchased from tilibit nanosystems) in TE buffers containing MgCl_2_ (20 mM) and NaCl (5 mM). The mixture was annealed from 80 °C to room temperature in approximately 28 h to assemble origami structures with the aptamer and complementary strands. The origami structures were purified using centrifuge filters with the molecular weight cutoff size of 100 kDa following the instruction provided by the manufacturer (Millipore). The concentration of origami structures was calculated by measuring the absorbance at 260 nm using the extinction coefficient of 1.3 × 10^8^ M^–1^ cm^–1^.

### Assembly of DNA Origami-Gold Nanorods

Gold nanorods (AuNRs) were synthesized following the protocol adopted from the literature.^28,46^ For assembly of AuNRs of DNA origami templates, thiolated DNA strands (purchased from Biomers) were first attached to AuNRs using the procedure described in previous literature.^47,48^ The free thiol-DNA was washed away by centrifugation at 7k rcf for 30 min for 4 times. The DNA strands on the AuNRs hybridized with the extended sequence of the staple strands to anchor the AuNRs on the origami. The AuNR-DNA and origami were mixed with 15:1 ratio and annealed from 40 °C to room temperature. To purify the samples, the origami-AuNRs were loaded into a 0.7% agarose gel with 13 mM MgCl_2_. After running the gel electrophoresis at 80 V for 3 h with ice cooling, the origami-AuNR band was cut and extracted. The concentration of origami-AuNR constructs was calculated by measuring the absorbance at maximum peak (at ∼650 nm) with an estimated extinction coefficient of 3.8 × 10^9^ M^–1^ cm^–1^.

### CD Measurements

The origami-AuNRs, which employed a pair of aptamer and complementary strand, were incubated in 70 μL PBS buffers [supplemented with MgCl_2_ (5 mM)] with/without analyte overnight at room temperature with shaking. The analyte concentrations of ATP, glucose, and thrombin were 1 mM, 100 mM, and ∼170 nM (20 units mL^–1^), respectively. The control analytes (GTP/CTP/UTP, fructose, and protein markers of different sizes) were used at the same or higher concentration as the target analytes. The CD spectra and extinction spectra were measured using a Jasco J-1500 CD spectrometer.

## Supporting Information Available

Materials and methods, equations and their derivations, data analysis, and other supporting data (PDF)
