# Supplementary material for: Characterizing Aptamers with Reconfigurable Chiral Plasmonic Assemblies
Source: Langmuir. 2022 Feb 25;38(9):2954–60. doi: 10.1021/acs.langmuir.1c03434 (PMC8908738; doi:10.1021/acs.langmuir.1c03434)
Supplement: Supplementary file 1 — la1c03434_si_001.pdf [file la1c03434_si_001.pdf]

Supporting Information for:

Characterizing aptamers with reconfigurable chiral plasmonic assemblies

Yike Huang[1,*], Minh-Kha Nguyen[1,2,3], Vu Hoang Nguyen[1], Jacky Loo[1], Arttu J. Lehtonen[1,4], Anton Kuzyk[1,*]

[1] Department of Neuroscience and Biomedical Engineering, School of Science, Aalto University, FI-00076 Aalto, Finland

[2] Faculty of Chemical Engineering, Ho Chi Minh City University of Technology (HCMUT), 268 Ly Thuong Kiet St., Dist. 10, Ho Chi Minh City 700000, Vietnam

[3] Vietnam National University Ho Chi Minh City, Linh Trung Ward, Thu Duc Dist., Ho Chi Minh City 700000, Vietnam

[4] Department of Electrical Engineering and Automation, School of Electrical Engineering, Aalto University, FI-00076 Aalto, Finland

Contents

Section 1. Materials and methods 2

1.1. Materials 2

1.2. Design of DNA origami structures 3

1.3. Assembly of DNA origami-based chiral probes 11

1.4. Microscopy and spectroscopy characterization 11

Section 2. Analytical model and data analysis 13

2.1 Analytical model 13

2.2 Data analysis 16

Section 3. Design considerations and benefits of plasmonic chiral probes for aptamer characterization 23

Section 4. Supporting data 24

Section 1. Materials and methods

1.1. Materials

DNA scaffold strands (p7650) were purchased from tilibit nanosystems; staple strands from ThermoFisher; thiol modified T_{16} DNA strands from Biomers; other DNA strands from IDT. Buffers and other chemicals were purchased from Fisher Scientific or Sigma-Aldrich. All reagents are commercially available and were used without any further purification. Type I ultrapure deionized (DI) water from the Milli-Q system was used for all experiments.

Table S1. Sequence of aptamers used in this study

Name	Sequence	Length (nt)
ATP DNA aptamer[1]	ACC TGG GGG AGT ATT GCG GAG GAA GGT	27
Glucose DNA aptamer[2]	ACGACCGTGTGTGTTGCTCTGTAACAGTGTCCATTGTCGT	40
Thrombin DNA aptamer[3]	GGTTGGTGTGGTTGG	15

1.2. Design of DNA origami structures

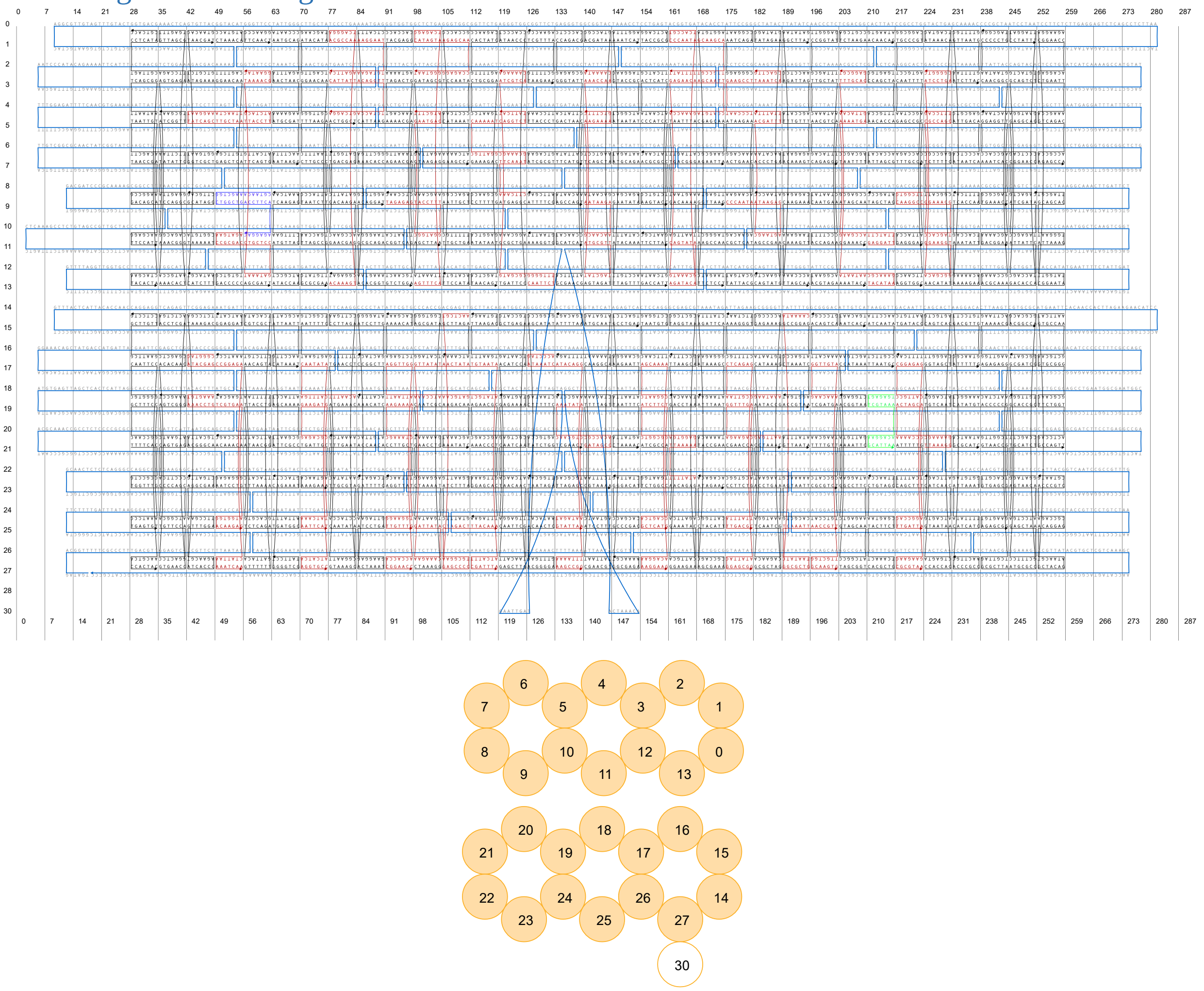

Figure S1. Scaffold/staples layout of the DNA origami structures designed using cadnano.[4]

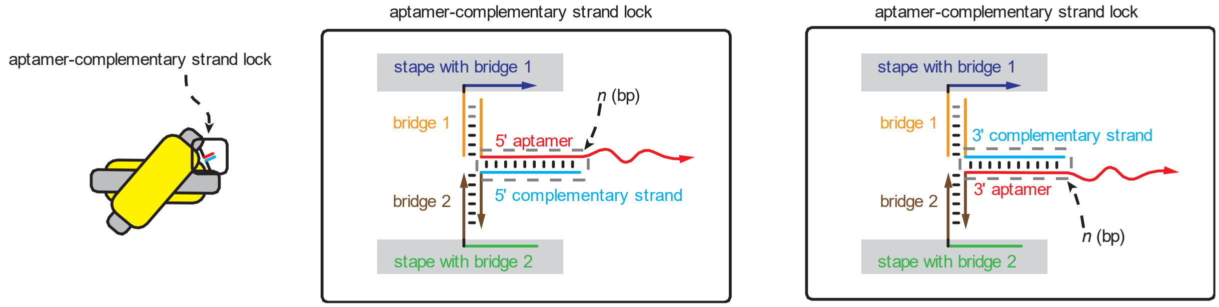

Figure S2. Design of aptamer - complementary strands molecular lock.

Table S2. Core strands of origami.

Start	End	Sequence
4[34]	13[34]	AATAATTTCAGCGGCTACGAATACACTA
8[90]	6[77]	AGGTCCGGATATTCTGACGAGGATGGTT
24[146]	18[133]	AGTAATCTTTTAGTCTA GAAAAAGCCTA
25[161]	16[161]	GAAATACCATTGCATTAAGCAGCCTTTA
26[146]	16[133]	TATTAATCAAGGCATAAAAATTTTTAGA
16[104]	14[91]	TCATAGGTCTGAGAAAAACATCACGAAT
22[146]	23[146]	TCTTTAATGCGCAGTTAGAGCCGTAAAA
25[203]	17[216]	GTAGCAAATCGGCCCCATAAATTAATGC
18[34]	27[34]	GGGGTGCCAATTCCAACGTCACCACTAC
13[35]	1[48]	AAACACTTGAGTTTGTTAGCGTAACGAT
4[223]	13[223]	AGCCTAACAATTTTTTCAACCAGGTGGC
25[245]	16[245]	GAGCTAAGCTTTCCGCGATCGGGCGATT
2[139]	0[126]	GCGAGAGCCAGACGCACCCTCAGAACCG
26[62]	16[49]	GATTACAAACAGTACTTCTGTAAATCCC
4[76]	13[76]	CGTTGGGGGAACAATTGTATCGCGCGAA
4[118]	13[118]	TGAATCCCTGCGGATAGCTCATAACAGT
26[230]	16[217]	CTGAGTAGGTAGCTCAGGGTTTTTTCAA
23[203]	18[203]	GGCCTTCAAGAGTCACGGTAACTGGAGC
27[140]	15[153]	CGAACGTATGATACATGCAATGCCTGAG
9[245]	4[238]	ATCGATATAGAGCCGAGGCAGGAATGGAAACTAAC
2[202]	13[195]	TTTAGCGCCGGTATGAGGGTTGATATAATTAGCAA
12[174]	12[182]	TAAGATCAAGCCAACGCTCATAGCCGAAAGAACTG
27[98]	16[105]	CTAAAGGCGGTGGGAGCGATAGCTTAGAATCAAAA
25[140]	22[147]	ATCCTTTGCCCGACCACGACCGGGACATCTATTAG
1[49]	12[42]	CTAAACAATGTACCGTAACACCATCTTTAATACGT
23[175]	19[195]	CCTTCTGACCTGAAAAATGGAAATACCGACCGTAA
18[132]	21[139]	CTAATAGCTTTTTCAGTGCCATATCTGGTCGAACT
6[97]	8[91]	TAAATTGGGCTTGAAAACACCAGAACGACTCCAAC

27[77]	16[84]	GTAAAGCCCTTGAACCTTAGAATCCTTGGACTACC
5[84]	9[90]	CATTAAGAAAACGATTAATCATAAGGGAACAGGAT
4[202]	13[209]	AATAAACTTGCTATACGGAATACGTAGAAAATACA
22[83]	22[91]	AATATACTTTGAATACCAACACCTTGCTTTTAACG
19[196]	23[202]	TCGATGATGTCCATCACGCTGAGTAATTCGCGTCT
2[181]	13[174]	CGGGAGGAATCAGACCGGAATAGGTGTATTCTCCT
6[153]	9[160]	AATAGATATGCAGAACGCGCCTCTGTCCAAGTACC
25[77]	18[84]	CAATATACGGAATTTGAACCTCCGGCTTACATTTA
21[196]	21[188]	TTTTGTTCGCCATCAAAAACGTAAGAATCCTAAAC
1[154]	13[160]	TACCGCGACTCAGGAGGTTTATTAGTTTGACCATT
22[209]	21[195]	ATAGGAAAAAAATTCGATTGTATAAGCAAATATTTAGTTAATA
7[175]	7[174]	ACTGAACACAAGAATTGAGTAAAGTAATTGACGGGAGAATTA
19[168]	22[168]	ATTTAATTAAAACATACCGAACGAACCAACGTGGCACAGACA
24[111]	20[98]	GTATTTCATCTTTAGGAATTGAGGAAGGGAACCTCGAAAAAT
27[56]	17[69]	GTTTTTTTCACATACGTCGCTATTAATTAACCTTGCATAAAT
4[139]	11[139]	CTTATCATTCCAAATTACCCTGACTAACCCAACATGCATCAT
22[251]	21[237]	GGATAGGTGCATCTCCAGCTTTCGGTTGATAATCAGCGCATC
7[140]	7[139]	TCAGCTAAAGTCCTGATTATAGTCAGAAATCGCGTTTCATGT
23[77]	20[77]	TTGCGTACCTACCAATGAAACAAACATCATGTTACAAAATCG
22[62]	22[63]	TTACATCGGGAGAGAATCCTGTTTAAAACAGAAATGTACCTT
12[195]	8[182]	ACCCAAACAAAGTTTAAGAAACAAGAAACAGAGAGATAACCC
3[238]	1[251]	CAACGGCGCCAAAGACGCAAAGACACCATTAGGATCCTATTT
4[160]	11[160]	ATCAATAACTCATCGTCAATAACCTGTTATACAAATTCTTAC
16[160]	26[147]	TTTCAACTAATGTGCCTGGAGTGACTCTGGCGAGAAAAACGT
20[48]	20[49]	TGCATTAACGGGCAACAAACAATAACGGATTCATTTCCCAGC
11[35]	4[35]	AAACGGGTAAAAATCGGTGTACAGACGGATCGGTTTGCGAAT
17[238]	15[251]	AGAGAGGTCGTTAGGCGCTTAATGCGCCGCGCACGACGGCCA
6[48]	6[49]	GGTGAATGGTCGCTGAGCTCATTCAGTGATCATTGTGTTCGA
7[28]	8[42]	TAACCGAAAGGCCGGACAGCATCCAGGCGCATAGGCTTGCAG
16[153]	21[153]	GCAAGGAAAGAATTATAATTATAATTTCAGTATTACTAAAAC

10[153]	9[139]	CATATTTGAATATAAGACGACGACAATAAACAATAAGCCAGT
13[119]	3[132]	TGATTCCCCACCCTATAACCCTCGTTTAGCTTTTGTAAGAAC
16[244]	26[231]	AAGTTGGTAAAACGACTTAAGTGTCCTTACCCGCCAACTTGC
7[112]	8[126]	GAAAGACGCGAACCCCTTTTGATGAGGCATTTTCGATTCGAG
27[119]	14[133]	GAGCTTGTGCCATCGCTGAGAAGCATATATTTTAACGACAGT
25[224]	22[224]	GTAATAACATCATCTTTTATAATTAAATGGTGTAGATTCAGC
12[181]	1[195]	GCATGATTATTACGCAGTATGGTATAGCTATAGAAGGCTTAT
2[76]	1[62]	TCAGTTGATACATATAGCAAGCCCAATAGGAACCCTTCAACT
27[35]	14[49]	GTGAACCACTCTGAACCTCGATAAAGACGGAGGATAATCATT
22[125]	24[112]	ATCAACACAATCAACGCTGAGAGCCAGCAGAACGCTTTAGAA
6[76]	12[77]	TAATTTCTTTAAGATGACCAATTAGCCGGAACGAGCGGAGAT
7[70]	5[83]	CTTGCCCATTACCCTAATCTTGACAAGAACCGAACACTGGCT
18[244]	25[244]	AATCTACAAAGGCTCATATGTACCCCCGGAAGTGTAGAGCGG
27[224]	17[237]	CCACCACAGTGCTGCAGTCACGACGTTGGTAACGCATTTTTG
2[97]	13[97]	AGTAAAATACGAGGCACCACCCTCATTTACACGGTGTCTGGA
12[41]	0[28]	AATGCCAAGTGAGATTCCAGACGTTAGTCCTCATACGTCACC
13[224]	3[237]	AACATATTCAGTACATAAACAGTTAATGGTTTTAAATCTTAC
14[174]	23[174]	CTATTTAAGCGAAACGCCAGCCTACATTCATTGGCATAGAAC
10[125]	10[126]	AGGCAAAGAGGTCAATATAATGCGCTGAAAAGGTGGTAATTT
2[118]	12[105]	AGTTTTGCACTATCCAGAACCGCCACCCTTCCATAACATGTT
8[250]	11[244]	AGACTGCGGAACCCCCTCAGAGCCACCGGAGGTTAGCAAAAGACGGAA
3[133]	1[153]	GGGTATTATTTTCAGCGAACGAGTAGATGTACCGCACGATAAAAATCAT
25[56]	18[49]	TCTCAGATGATGGCATTATTTGCACGTGTTACCTGTTTTTAATGAGCAT
23[91]	21[111]	AGGTTATCTAAAATTGAATAACAATCGCAAGACAAAGCAAATAAATATC
11[105]	7[111]	TTGCTGATTTTTGCAATTGCTAGACCGGAAGCAAAGTAAGAGGAAGCCC
2[195]	7[202]	AACCTCCAGATTAGAGCCATATTTGTTTAGAGAATACAAAGTCAGAGGG
27[161]	18[168]	GGAAGAACGCTCGCTAGGTAAAGATTCAGGGAGAAATAAAGCATAAGAA
4[237]	9[244]	GAGCGTCATTGACAACCCTCAATAATCAAAATCACTAGCGCGTGAAACC
21[238]	18[245]	GTAACCGTCACGTTGTGAGCGAGTAACAATCCTGAGCACCGCTGTTGGG
21[28]	18[35]	TTTTCACCCGCCTGTGGTTTGCCCCAGCATCGGCAAGTCGGGAAAGCCT

2[216]	11[223]	TGAGTGCCCAGCTATTTGCCAAACACCAGAGCCGCATTACCAGAGGGAG
16[83]	26[63]	TTTTTTGAGTGAATAATTTTCTCGGCTGACGCATTGGGGTCGTATTCCT
27[203]	22[210]	TAGCGGTCACGCTGTCAAACTTACTTCTCGAGTAACTGTAGCTTAACCA
15[252]	21[258]	GTGCCAAGCTGCAAGTGCGGGGCGCAACTTCTGGTACTCCAGGCCAGTT
4[97]	11[104]	AAACAGTTAGACTGTGCAACTAAAGTAAGCGCAGACGGTCAGAGCTTAA
18[69]	23[76]	ATTACCTAGCAAAAGCGAATTATTCGCCTGATTGCAGTAACAAAAGAAA
7[182]	9[174]	ACCCTGAAACATAAAAACAGGATAAGAAAGAACAGTAGGGCTGGTTAAG
21[154]	18[161]	ATCGCCATGAATGGTCTGGCCAACAGAGAGATTCAGACCTAATAAACAC
2[160]	10[154]	TTCATCGTAGGAACGTACCGCATCGGCTATAATATCCCATCCGAATCGC
0[237]	8[231]	GTTTTGCAAAAGAAACAAAAGAAATATTTCACCAGTCACCAATTTTCAT
7[203]	11[209]	TAATTTTAGCAGCCTTTACAGAACGTCACCCTTTTACCAGAAGGAAACC
14[258]	22[252]	AATTCATGCTACAGATAACGTACAGGAGCGCCAGAACCCGTCCGTAATG
25[35]	14[28]	TGTTCCAGGACTCCACACAACCGAGCTCGAATTCGGCTTGTTCCTCCTG
25[182]	14[175]	TCAATCGCAGAACACATAAAGTGTAATACTTTTGCAAAGGGTTTCTAAT
1[252]	7[258]	CGGAACCACAGGAGCTGAATTAAAGCCAGTCAGACACCGCCAAGAGCCA
2[223]	1[216]	AGTGCCTTGCCCGTCAGGCGGATAAGTGCCGTCGATCTAAGAACAACAG
8[230]	7[223]	CGGCATTTCTTTTCGAGCCGCCACCAGAACCACATATTAGCGTTTGCCA
22[41]	25[34]	CCCTTCACAGTGAGATGAATCGGCCAACGCTTTCCAAATCCCTGAGTGT
14[48]	22[42]	TCTCCGAATCACCCAGAACGTGTTTGGATTCCGAAAGGCGAACTGATTG
11[245]	0[238]	ATTATTCTACCAGCGCAGTCTTGTACTGGTAATAACCCCCTGTAGCGGG
14[132]	22[126]	GCGGCCCACGGGGAGTTTGAGACAACTCAATACATTAATAGATTGGCAA
21[217]	18[224]	ATTTTTGTCATTTTCAGCTTTCATCAACATCAGTGTGTCAATATCAGGT
1[63]	7[69]	AATGCAGAGATTTAAACTAACAAGAAAAATGCGATAACTTTAAATAAGG
27[182]	18[189]	GCGCTAGACCCCGCGAGAAAGGCCGGAGATGACCCCTAAATCAGGTGAT
25[119]	14[112]	CAATTCGTAACATTACATCCATCAATAGTGAATTTTTAAGACTGTAAGC
14[90]	22[84]	ATAGGGGACTAAATGAAGGAGATCCTGAGTTAGAAGATTTTCTCAGATG
12[104]	6[98]	TTAAATAGATAGCGTCCAATACCCTCAACATAAATATCAAAAAGATTAG
8[41]	11[34]	GGAGTTATATATTCTTCTTAAACAGCTTTAATTGTAACGAGGTTCCATT
9[161]	6[154]	GACAAAATAATTGATAATTTACGAGCAAGAAGCGCATTAGTTTATCAAC
21[112]	18[119]	AAACCCTGTTGAAAGGAGCACTAACAACTTGAGGAGAGAAAATAGTAGC

2[55]	13[62]	CAGTTTTGTCGTCTATAGAAAGGAACAATGTGTCGGACCCCCAGCGATT
0[258]	8[251]	AGAAGGACGGAATATATGGTTATTAAAGTGGGAATGCAGCACTTAGCGTC
13[63]	8[63]	ATACCAAATCGCCTATGTTACCTTTGAATCAAGAGAAATCAA
15[217]	15[209]	TGATACCAATTGTCAACCTTATGACAATAATCACC
11[140]	2[140]	ATGCGTTTAGCTATAAACCAACAAAATA AAA AAA AAA A
18[160]	27[160]	CGGAATCAGCAAAAACAGGAAAAGGAAG AAA AAA AAA A
20[97]	25[97]	CTAAAGCAAGAAAATGGAAGGTTGTTTG AAA AAA AAA A
3[77]	4[77]	CATTATTACAGGTTTCTACCAGTCAGGA AAA AAA AAA A
4[181]	2[182]	TCCCAATGAAGCCTTAAATCACGACTTG AAA AAA AAA A
9[140]	4[140]	AATAAGAAACAACGAAGAAAAGTCTTTC AAA AAA AAA A
20[76]	25[76]	CGCAGAGGAAGATGTATCAAAAATTCAT AAA AAA AAA A
18[202]	27[202]	AAACAAGGGTTGTATTGCTGGGCAAGTG AAA AAA AAA A
18[223]	27[223]	CATTGCCCGGAGAGGAAGAACCGCGTAA AAA AAA AAA A
11[224]	2[224]	GGAAGGTGGCGACAATCCTGACGGGGTC AAA AAA AAA A
18[48]	25[55]	AAAGTGTAAACCTGTCGTGAAATGGTGGACAAGAG AAA AAA AAA A
16[132]	27[139]	ACCCTAGATAAATCATACAGGTTTAAAAAAGCCGG AAA AAA AAA A
9[91]	4[98]	TAGAGAGTACCTTTGGATGGCGAATGACATGCTTT AAA AAA AAA A
13[210]	2[203]	TACATAAGAAACGCAATAATATTTGCACGAGGCGT AAA AAA AAA A
18[83]	27[76]	ACAATTTCATTTGACAATATAATCATCAAGGTGCC AAA AAA AAA A
11[210]	4[203]	GAGGATTTTATCTTACCGAAGAAAATGAGTTACAA AAA AAA AAA A
20[188]	25[181]	AATTGAGCAGAAGAGGTTTGATTATTTATTGACGC AAA AAA AAA A
13[126]	2[119]	CAATTCTTTTGGGGCGCGATGATCGTCACAAAAGA AAA AAA AAA A
14[195]	25[202]	CAAAATAGGCGCTGTAATATCTCAAATTAACCGTT AAA AAA AAA A
18[188]	27[181]	AAATAAGGCGTTAACTCAGAGATATTACGGAGCGG AAA AAA AAA A
14[111]	25[118]	AACTCGTGAGCCCCTGCGGAACTAGACTTTACAAA AAA AAA AAA A
16[48]	27[55]	CGGGTACATACGAGCCGGAGACTATTAAAAATCAA AAA AAA AAA A
9[175]	4[182]	CCCAATAATAAGAGAGTAAGCACGATTTTTATTTA AAA AAA AAA A
21[140]	25[139]	GATAGCCACACCGCCTGCAACAAATATAAATAGATGTATTAA AAA AAA AAA A
10[55]	2[56]	AGATGAACCGCGACCTGCTCCGATAAATTAAAACGGGAATAC AAA AAA AAA A
13[98]	2[98]	AGTTTCATCAGAGCCATAGTAAGAGCAACCAGAGGGGGTAAT AAA AAA AAA A

4[55]	4[56]	TTAACTAAAGGAATTATCAGCTTGCTAATTACCTTATCTACG AAA AAA AAA A
11[161]	4[161]	CAGTATAGCAAATGGAGAACAAGCAATTCCATGTAGAAACCA AAA AAA AAA A
21[224]	25[223]	TTAAAGGGAAAAGCCCCAAAAACTAGCAAGGCCACTTGATTA AAA AAA AAA A
13[77]	2[77]	ACAAAGTTCAGGGAACGCCAAAAGGAATTGTAGAAAGATTCA AAA AAA AAA A
13[161]	2[161]	AGATACATCACCGTCCCAATAGCAAGCATTGCCGTTTTTATT AAA AAA AAA A
8[223]	4[224]	TTCGGTCCAAGGCCGGAAACGTAGCACCCGCCAGCTTTCCAG AAA AAA AAA A
18[118]	27[97]	ATATGCTGATGCAAATCCAAAATTAATTAGGTTGGACCACCACGGAACC AAA AAA AAA A
8[125]	4[119]	CTTCAAATTCAAATGCAAAGCGGATTGCCAAAAATCAGGTCTTATTCAT AAA AAA AAA A
22[167]	25[160]	ATATTTTTTAAAAAGAGGTGAGGCGGTCATCTTCTCCAGTCAGCTCATG AAA AAA AAA A
25[98]	27[118]	GATTATACAAAGAAGTTATATAACTATATGTAATAATCATTTCGATTTA AAA AAA AAA A

Table S3. Bridge and lock strands.

Name	Sequence
Staple with bridge 1	GTT CGC TCG CTA G TT CGTAACAAAGCTGGCTGGCTGACCTTCAAGAGGAC
Staple with bridge 2	TGAGAGTTCGTAAAACAGGAAGCATTAA TT CTC TGC TCA CTC T
5'template-bridge	CTA GCG AGC GAA C ATA GTG AGT TCA ACT TGG ACC AC
5'tempCS8, (*n*=8)	GAA CTC AC ATA AGA GTG AGC AGA G
5'tempCS9, (*n*=9)	TGA ACT CAC ATA AGA GTG AGC AGA G
5'tempCS10, (*n*=10)	TTG AAC TCA C ATA AGA GTG AGC AGA G
5'tempCS11, (*n*=11)	GTT GAA CTC AC ATA AGA GTG AGC AGA G
5'tempCS12, (*n*=12)	AGT TGA ACT CAC ATA AGA GTG AGC AGA G
5'tempCS13, (*n*=13)	AAG TTG AAC TCA C ATA AGA GTG AGC AGA G
5'tempCS14, (*n*=14)	CAA GTT GAA CTC AC ATA AGA GTG AGC AGA G
competitor strand (S)	CAA GTT GAA C
5'ATP aptamer-bridge	CTA GCG AGC GAA C TTT ACC TGG GGG AGT ATT GCG GAG GAA GGT
5'ATPCS12, (*n*=12)	ACT CCC CCA GGT TTT AGA GTG AGC AGA G
5'ATPCS11, (*n*=11)	CT CCC CCA GGT TTT AGA GTG AGC AGA G

5'ATPCS10, (*n*=10)	T CCC CCA GGT TTT AGA GTG AGC AGA G
5'ATPCS9, (*n*=9)	CCC CCA GGT TTT AGA GTG AGC AGA G
3'ATP aptamer-bridge	ACC TGG GGG AGT ATT GCG GAG GAA GGT TTT AGA GTG AGC AGA G
3'ATPCS12, (*n*=12)	CTA GCG AGC GAA C TTT ACC TTC CTC CGC
3'ATPCS11, (*n*=11)	CTA GCG AGC GAA C TTT ACC TTC CTC CG
3'ATPCS10, (*n*=10)	CTA GCG AGC GAA C TTT ACC TTC CTC C
3'ATPCS9, (*n*=9)	CTA GCG AGC GAA C TTT ACC TTC CTC
5'thrombin aptamer-bridge	CTA GCG AGC GAA C TTT GGT TGG TGT GGT TGG
5'thrCS11, (*n*=11)	C CAC ACC AAC C TTT AGA GTG AGC AGA G
5'thrCS10, (*n*=10)	CAC ACC AAC C TTT AGA GTG AGC AGA G
5'thrCS9, (*n*=9)	AC ACC AAC C TTT AGA GTG AGC AGA G
5'thrCS8, (*n*=8)	C ACC AAC C TTT AGA GTG AGC AGA G
5'thrCS7, (*n*=7)	ACC AAC C TTT AGA GTG AGC AGA G
3'thrombin aptamer-bridge	GGT TGG TGT GGT TGG TTT AGA GTG AGC AGA G
3'thrCS11, (*n*=11)	CTA GCG AGC GAA C TTT CCA ACC ACA CC
3'thrCS10, (*n*=10)	CTA GCG AGC GAA C TTT CCA ACC ACA C
3'thrCS9, (*n*=9)	CTA GCG AGC GAA C TTT CCAACCACA
3'thrCS8, (*n*=8)	CTA GCG AGC GAA C TTT CCAACCAC
3'thrCS7, (*n*=7)	CTA GCG AGC GAA C TTT CCAACCA
5'glucose aptamer-bridge	CTA GCG AGC GAA C TTT ACG ACC GTG TGT GTT GCT CTG TAA CAG TGT CCA TTG TCG T
5'gluCS12, (*n*=12)	ACA CAC GGT CGT TTT AGA GTG AGC AGA G
5'gluCS11, (*n*=11)	CA CAC GGT CGT TTT AGA GTG AGC AGA G
5'gluCS10, (*n*=10)	A CAC GGT CGT TTT AGA GTG AGC AGA G
5'gluCS9, (*n*=9)	CAC GGT CGT TTT AGA GTG AGC AGA G
5'gluCS8, (*n*=8)	AC GGT CGT TTT AGA GTG AGC AGA G
3'glucose aptamer-bridge	ACG ACC GTG TGT GTT GCT CTG TAA CAG TGT CCA TTG TCG T TTT AGA GTG AGC AGA G
3'gluCS12, (*n*=12)	CTA GCG AGC GAA C TTT ACG ACA ATG GAC
3'gluCS11, (*n*=11)	CTA GCG AGC GAA C TTT ACG ACA ATG GA
3'gluCS10, (*n*=10)	CTA GCG AGC GAA C TTT ACG ACA ATG G

3'gluCS9, (*n*=9)	CTA GCG AGC GAA C TTT ACG ACA ATG
3'gluCS8, (*n*=8)	CTA GCG AGC GAA C TTT ACG ACA AT
5'CS0, (*n*=0)	TTT AGA GTG AGC AGA G
3'CS0, (*n*=0)	CTA GCG AGC GAA C TTT

1.3. Assembly of DNA origami-based chiral probes

Fabrication of DNA origami structures with aptamer and complementary strands

To prepare the staple solutions, 156 core strands, 2 bridge strands, 1 pair of the aptamer and the complementary strand (lock strands) with the ratio of 1:1.5:2 were mixed. Ten microliter of staples (0.64 μM, 0.96 μM, and 1.3 μM for core, bridge, and lock strands, respectively) solution was mixed with 5 μL TE (10×) buffer, 5 μL scaffold p7560 (100 nM), 10 μL $MgCl_2$ (100 mM), 2.5 μL NaCl (100 mM), and 17.5 μL H_2O. The mixture was annealed from 80 °C to room temperature in approximately 28 h to assemble origami structures with the aptamer and complementary strands[1]. The origami structures were purified using centrifuge filters with the molecular weight cut off (MWCO) of 100 kDa following the instruction provided by the manufacture (Milipore). The concentration of origami structures was calculated by measuring the absorbance at 260 nm using the extinction coefficient $1.3 \cdot 10^8$ $M^{-1}cm^{-1}$. The purified origami structures were stored at 4 °C in DNA LoBind tubes after purification. To examine the origami structures, 10 μL samples were run through a 2% agarose gel in 0.5× TBE buffer with the supplement of 10 mM $MgCl_2$. The gel was run at 100 V for 4 h. The Sybrgold was used as the staining agent and the gel was imaged with the Bio-Rad Gel Doc XR system.

Assembly of DNA origami-gold nanorods

Gold nanorods (AuNRs) were synthesized following the protocol adopted from literature.[5,6] For assembly of AuNRs of DNA origami templates, thiolated DNA strands were first attached to AuNRs using the procedure described in the previous literature.[7,8] The free thiol-DNA was washed away by centrifugation at 7k rcf for 30 min for 4 times. The DNA strands on the AuNRs hybridized with the extended sequence of the staple strands to anchor the AuNRs on the origami. The AuNR-DNA and origami were mixed with 15:1 ratio and annealed from 40 °C to room temperature. To purify the samples, the origami-AuNRs were loaded into a 0.7% agarose gel with 13 mM $MgCl_2$. After running the gel electrophoresis at 80 V for 3 h with ice cooling, the origami-AuNRs band was cut and extracted. The concentration of origami-AuNRs constructs was calculated by measuring the absorbance at maximum peak (at ~650 nm) with an estimated extinction coefficient of $3.8 \cdot 10^9$ $M^{-1}cm^{-1}$. The purified origami-AuNRs constructs (see Figure S5) were stored at 4 °C.

1.4. Microscopy and spectroscopy characterization

Transmission electronic microscopy (TEM)

The origami-AuNRs constructs were imaged using FEI Tecnai F12 electron microscope operated at 120 kV. To deposit origami-AuNRs constructs on grid, 5 μL of the sample solutions was adsorbed onto a glow discharged carbon-film-coated copper grids for 8 min, followed by staining with a 1% uranyl formate solution containing 25 mM NaOH for 20 s.

Circular dichroism (CD) measurements

The origami-AuNRs, which employed a pair of aptamer and complementary strand, were incubated in 70 µL buffers (1×PBS sumplemented with $MgCl_2$ (5mM)) with/without analyte for overnight at room temperature with shaking. The concentrations of the origami-AuNRs constructs were between 15 and 50 pM. The analyte concentrations of ATP, glucose, and thrombin were 1 mM, 100 mM, and ~170 nM (20 units mL^{-1}), respectively. The control analytes (GTP/CTP/UTP, fructose, protein markers of different sizes) were used at the same or higher concentration as the target analytes. The CD spectra and extinction spectra were measured using Jasco J-1500 CD spectrometer.

1.5. Reproducibility

Affinity and specificity characterization workflow was repeated twice starting from staples, scaffold and AuNRs. The outcomes of two independent experiments were consistent.

Section 2. Analytical model and data analysis

2.1 Analytical model

Concentrations at equilibrium

$$\mathrm{A + C \rightleftharpoons AC} \qquad (reation\ 1, \Delta G_1^{\circ}, K_{\mathrm{D1}}),$$
$$\mathrm{A + B \rightleftharpoons AB} \qquad (reation\ 2, \Delta G_2^{\circ}, K_{\mathrm{D2}}),$$
$$\mathrm{AC + B \rightleftharpoons AB + C} \qquad (reation\ 3, \Delta G_3^{\circ}, K_{\mathrm{D3}}),$$

where, A, B and C are aptamer, analyte, and complementary strand, respectively. Of note, although the replacement reaction (reaction 3) is present in the system, K_{D1} (reaction 1) and K_{D2} (reactions 2) are sufficient to determine the concentration of each spices at equilibrium (Table S4). In Table S4, a_0 stands for the total input local concentration of A strand, which is equal to the total input local concertation of C strand; b_0 is the total input bulk concentration of analyte (B). As the amount of B (≥10 pmole) is much larger than the amount of A (<1 fmole), the concentration of analyte at equilibrium approximately equals to the initial concentration b_0. For a fixed aptamer and complementary strand with the hybridization length of n base pair, x_n is the concentration of the AC hybrid in the absence of analyte; y_n and z_n are the concentrations of AC hybrid and the AB aptamer-analyte complex in the presence of the analyte, respectively. The concentrations of the other species were calculated from the mass conservation.

Table S4. The concentration of each spices at equilibrium.

	[A]	[B]	[C]	[AB]	[AC]
**Equilibrium state 1 (without analyte)**	$a_0 - x_n$	0	$a_0 - x_n$	0	x_n
**Equilibrium state 2 (with analyte)**	$a_0 - y_n - z_n$	b_0	$a_0 - y_n$	z_n	y_n

To compare the two equilibrium states of the hybridization between aptamer (A) and complementary strands (C) in the presence and absence of analyte molecules (B), the following equations were applied:

$$K_{\mathrm{D1}} = \frac{[\mathrm{A}]\cdot[\mathrm{C}]}{[\mathrm{AC}]} \qquad (S1)$$

$$K_{\mathrm{D2}} = \frac{[\mathrm{A}]\cdot[\mathrm{B}]}{[\mathrm{AB}]} \qquad (S2)$$

Substitute the concentration into the equation S1 and S2. therefore,

$$K_{D1}(n) = \frac{(a_0 - x_n)^2}{x_n} \qquad (S3)$$

$$K_{D1}(n) = \frac{(a_0 - y_n - z_n)\cdot(a_0 - y_n)}{y_n} \qquad (S4)$$

$$K_{D2} = \frac{b_0 \cdot (a_0 - y_n - z_n)}{z_n} \qquad (S5)$$

Solve x_n, y_n, and z_n:

$$x_n = a_0 + \frac{K_{D1}(n)}{2} - \frac{\sqrt{4a_0 K_{D1}(n) + K_{D1}^2(n)}}{2} \quad (S6)$$

$$y_n = \frac{\frac{K_{D1}(n)}{K_{D2}}(K_{D2}+b_0)+2a_0-\sqrt{\left(1+\frac{2b_0}{K_{D2}}+\frac{b_0^2}{K_{D2}^2}\right)K_{D1}^2(n)+\left(4a_0+\frac{4a_0 b_0}{K_{D2}}\right)K_{D1}(n)}}{2} \quad (S7)$$

$$z_n = \frac{K_{D1}(n)}{K_{D2}} \cdot b_0 \cdot \frac{y_n}{(a_0 - y_n)} \quad (S8)$$

Define β as the ratio of the AC concentrations. For a fixed aptamer and its complementary strand with the hybridization length of *n* bp, the ratio (β_n) of the AC concentrations in the presence and absence of the analyte:

$$\beta_n = \frac{y_n}{x_n} = \frac{K_{D1}(n)+b_0\frac{K_{D1}(n)}{K_{D2}}+2a_0-\sqrt{\left(1+\frac{2b_0}{K_{D2}}+\frac{b_0^2}{K_{D2}^2}\right)\cdot K_{D1}^2(n)+\left(4a_0+\frac{4a_0 b_0}{K_{D2}}\right)\cdot K_{D1}(n)}}{2a_0+K_{D1}(n)-\sqrt{4a_0 K_{D1}(n)+K_{D_1}^2(n)}} \quad (S9)$$

Circular dichroism signals at equilibrium

The measured circular dichroism signal (CD) at the minimum peak wavelength (~620nm), measured absorption (Abs) at the maximum peak wavelength (~650 nm), and the normalized CD signal (*N*) can be expressed in terms of concentrations and molar optical coefficients as:

$$CD = c_{\text{closed}} \cdot \varepsilon_{\text{CD}}^{\text{closed}} \cdot l + c_{\text{open}} \cdot \varepsilon_{CD}^{\text{open}} \cdot l \quad (S10)$$

$$Abs = \left(c_{\text{closed}} + c_{\text{open}}\right) \cdot \varepsilon_{\text{Abs}} \cdot l \quad (S11)$$

$$N = \frac{CD}{Abs} = \frac{c_{\text{closed}} \cdot \varepsilon_{\text{CD}}^{\text{closed}} + c_{\text{open}} \cdot \varepsilon_{\text{CD}}^{\text{open}}}{\left(c_{\text{closed}} + c_{\text{open}}\right) \cdot \varepsilon_{\text{Abs}}} \quad (S12)$$

where c_{closed} and c_{open} are the concentrations of origami-AuNRs constructs in the closed and open configuration, respectively; $\varepsilon_{\text{CD}}^{\text{closed}}$ and $\varepsilon_{\text{CD}}^{\text{open}}$ are the molar CD of origami-AuNRs constructs in the closed and open configuration, respectively; l is optical path; ε_{Abs} is the molar extinction for the origami-AuNRs constructs, which is irrelative to the configurational state.

The CD, Abs, and N can also be written in terms of the fraction of origami-AuNRs constructs in the closed configuration (f). Thus,

$$CD = c \cdot f \varepsilon_{\text{CD}}^{\text{closed}} \cdot l + c \cdot (1-f) \cdot \varepsilon_{\text{CD}}^{\text{open}} \cdot l \quad (S13)$$

$$Abs = c \cdot \varepsilon_{\text{Abs}} \cdot l \quad (S14)$$

$$N = \frac{CD}{Abs} = \frac{f \cdot \varepsilon_{\text{CD}}^{\text{closed}} + (1-f) \cdot \varepsilon_{\text{CD}}^{\text{open}}}{\varepsilon_{Abs}} = \frac{f \cdot \left(\varepsilon_{\text{CD}}^{\text{closed}} - \varepsilon_{\text{CD}}^{\text{open}}\right) + \varepsilon_{\text{CD}}^{\text{open}}}{\varepsilon_{\text{Abs}}} \quad (S15)$$

where c is total concentration of origami-AuNRs constructs in the sample, i.e., $c = c_{\text{closed}} + c_{\text{open}}$

For the sample of all origami-AuNRs constructs in the open configuration, i.e., without the aptamers and the complementary strands:

$$f = 0$$

$$CD_{\text{open}} = c \cdot \varepsilon_{\text{CD}}^{\text{open}} \cdot l \quad (S16)$$

$$N_{\text{open}} = \frac{\varepsilon_{\text{CD}}^{\text{open}}}{\varepsilon_{\text{Abs}}} \quad (S17)$$

For the sample of all origami-AuNRs constructs in the closed configuration:

$$f = 1$$

$$CD_{\text{closed}} = c \cdot \varepsilon_{\text{CD}}^{\text{closed}} \cdot l \quad (S18)$$

$$N_{\text{closed}} = \frac{\varepsilon_{\text{CD}}^{\text{closed}}}{\varepsilon_{\text{Abs}}} \quad (S19)$$

From the eqs S15 and S17, define the relative normalized CD signal N^*:

$$N^* = N - N_{\text{open}} = f \, \frac{\left(\varepsilon_{\text{CD}}^{\text{closed}} - \varepsilon_{\text{CD}}^{\text{open}}\right)}{\varepsilon_{\text{Abs}}} \quad (S20)$$

As the configuration states of the origami-AuNRs construct correspond to the hybridization states of the aptamer-complementary stands, the fraction of closed constructs equals to the fraction of AC,

$$f = \frac{c_{\text{closed}}}{c} = \frac{[\text{AC}]}{a_0} \quad (S21)$$

For a fixed aptamer and its complementary strand with the hybridization length of *n* bp, the fraction of closed origami-AuNRs constructs (f_n) in the absence and presence of the analyte:

$$f_n^{b_0=0} = \frac{x_n}{a_0} \quad (S22)$$

$$f_n^{b_0} = \frac{y_n}{a_0} \quad (S23)$$

From equation S20, for a fixed aptamer and its complementary strand with the hybridization length of *n* bp, the relative normalized CD signal (N_n^*) in the absence and presence of the analyte:

$$N_n^{*(b_0=0)} = N_n^{b_0=0} - N_{\text{open}}^{b_0=0} = \frac{f_n^{b_0=0}\left(\varepsilon_{\text{CD}}^{\text{closed}} - \varepsilon_{\text{CD}}^{\text{open}}\right)}{\varepsilon_{\text{Abs}}} \quad (S24)$$

$$N_n^{*(b_0)} = N_n^{b_0} - N_{\text{open}}^{b_0} = \frac{f_n^{b_0}\left(\varepsilon_{\text{CD}}^{\text{closed}} - \varepsilon_{\text{CD}}^{\text{open}}\right)}{\varepsilon_{\text{Abs}}} \tag{S25}$$

Therefore,

$$\frac{N_n^{*(b_0)}}{N_n^{*(b_0=0)}} = \frac{N_n^{b_0} - N_{open}^{b_0}}{N_n^{b_0=0} - N_{open}^{b_0=0}} = \left(\frac{f_n^{b_0}\left(\varepsilon_{\text{CD}}^{\text{closed}} - \varepsilon_{\text{CD}}^{\text{open}}\right)}{\varepsilon_{\text{Abs}}} \middle/ \frac{f_n^{b_0=0}\left(\varepsilon_{\text{CD}}^{\text{closed}} - \varepsilon_{\text{CD}}^{\text{open}}\right)}{\varepsilon_{\text{Abs}}} \right) = \frac{f_n^{b_0}}{f_n^{b_0=0}} = \frac{y_n}{x_n} = \beta_n \tag{S26}$$

2.2 Data analysis

To obtain ε:

Gibbs free energy of the DNA hybridization in the origami-AuNRs construct ($\Delta G_{\text{r}}^{\circ}$) may differ from the Gibbs free energy calculated from the mfold software ($\Delta G_{\text{theory}}^{\circ}$), which uses the data in the free solution. Thus, a correction coefficient ε was introduced,

$$\Delta G_{\text{r}}^{\circ} = \varepsilon \cdot \Delta G_{\text{theory}}^{\circ} \tag{S27}$$

To simplify further derivation, we define: $\frac{1}{\omega} = \varepsilon/RT$. R is the gas constant and T is the Kelvin temperature.

Therefore,

$$K_{\text{D1}} = e^{\frac{\Delta G_{\text{r}}^{\circ}}{RT}} = e^{\frac{\Delta G_{\text{theory}}^{\circ}}{\omega}} \tag{S28}$$

We used a template strand (A*) replacing the aptamer strand for calibration. The template strand-complementary strand hybridization length (n) was varied in a set of the origami-AuNRs constructs. Series of $\Delta G_{\text{theory}}^{\circ}(n)$ were obtained from mfold.[9] The normalized CD signals (N_n) were obtained from the measurements to calculate the fraction of the origami-AuNRs constructs in the closed configuration (f_n).

From equation S20,

$$N_n - N_{\text{open}} = \frac{f_n\left(\varepsilon_{\text{CD}}^{\text{closed}} - \varepsilon_{\text{CD}}^{\text{open}}\right)}{\varepsilon_{Abs}} \tag{S29}$$

Hence,

$$f_n = \frac{(N_n - N_{\text{open}}) \cdot \varepsilon_{\text{Abs}}}{\varepsilon_{\text{CD}}^{\text{closed}} - \varepsilon_{\text{CD}}^{\text{open}}} \tag{S30}$$

From equation S21,

$$f_n = \frac{x_n}{a_0} \quad (S31)$$

Therefore,

$$K_{D1}(n) = \frac{(a_0 - x_n)^2}{x_n} = \frac{(a_0 - a_0 f_n)^2}{a_0 f_n}$$

$$= a_0 \left(1 - \frac{(N_n - N_{\text{open}}) \cdot \varepsilon_{\text{Abs}}}{\varepsilon_{\text{CD}}^{\text{closed}} - \varepsilon_{\text{CD}}^{\text{open}}}\right)^2 \Big/ \left(\frac{(N_n - N_{\text{open}}) \cdot \varepsilon_{\text{Abs}}}{\varepsilon_{\text{CD}}^{\text{closed}} - \varepsilon_{\text{CD}}^{\text{open}}}\right)$$

$$= a_0 \ (\varepsilon_{\text{CD}}^{\text{closed}} - \varepsilon_{\text{CD}}^{\text{open}} - N_n \varepsilon_{\text{Abs}} + N_{\text{open}} \varepsilon_{\text{Abs}})^2 \Big/ \left((\varepsilon_{\text{CD}}^{\text{closed}} - \varepsilon_{\text{CD}}^{\text{open}})(N_n - N_{\text{open}})\varepsilon_{\text{Abs}}\right) \quad (S32)$$

Combined with equation S17 and S19,

$$K_{\text{D1}}(n) = \frac{a_0 \cdot \varepsilon_{\text{Abs}} \cdot (N_{\text{closed}} - N_n)^2}{(\varepsilon_{\text{CD}}^{\text{closed}} - \varepsilon_{\text{CD}}^{\text{open}}) \cdot (N_n - N_{\text{open}})} \quad (S33)$$

Therefore,

$$\Delta G^{\circ}_{\text{theory}}(n) = \omega \cdot ln K_{\text{D1}}(n) = \omega \cdot \ ln \frac{(N_{\text{closed}} - N_n)^2}{N_n - N_{\text{open}}} + \omega \cdot ln \frac{a_0 \varepsilon_{\text{Abs}}}{\varepsilon_{\text{CD}}^{\text{closed}} - \varepsilon_{\text{CD}}^{\text{open}}} \quad (S34)$$

In equation S34, N_{closed}, N_{open}, a_0, $\varepsilon_{\text{CD}}^{\text{closed}}$, $\varepsilon_{\text{CD}}^{\text{open}}$ and ε_{Abs} are constants (with $\varepsilon_{\text{CD}}^{\text{closed}} >> \varepsilon_{\text{CD}}^{\text{open}}$) and N_n depends on the hybridization length between the aptamer and the complementary strand (*n*, see Figure 1 in the main text and Figure S2). In principle, both N_{closed} and N_{open} can be obtained from experimental measurements. However, it is technically challenging to ensure that all the origami-AuNRs constructs at the closed configuration. Hence, *ω* was obtained by fitting $\Delta G^{\circ}_{\text{theory}}(n)$ dependence on N_n(with *n*={8,9,10,11,12,13,14}) with N_{closed} being another fitting parameter. From the fitting, the coefficient *ω* was determined as 3.76±0.373 kJ·mol^{-1} with R^2 =0.988, N_{closed} as 671.2, and *ε* value as 0.65 which is comparable with previous works.[10]

Define:

$$q(n) = ln \frac{(N_{\text{closed}} - N_n)^2}{N_n - N_{\text{open}}}, \ c = \omega \cdot \ ln \frac{a_0 \cdot \varepsilon_{\text{Abs}}}{\varepsilon_{\text{CD}}^{\text{closed}} - \varepsilon_{\text{CD}}^{\text{open}}} \quad (S35)$$

Then,

$$\Delta G^{\circ}_{theory}(n) = \omega \cdot q(n) + c \quad (S36)$$

The assumption that the Gibbs free energy of the system can be corrected by introducing *ε* was verified by the linearity between $\Delta G^{\circ}_{\text{theory}}(n)$ and the $q(n)$ (see Figure S3).

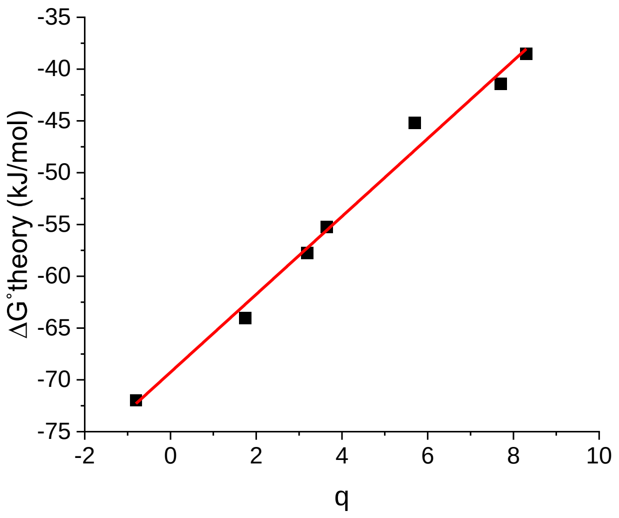

Figure S3. The linear fitting of $\Delta G^{\circ}_{\text{theory}}(n)$ dependence on $q(n)$

To obtain a_0:

The ratio of the relative normalized CD signal in the presence and absence of the analytes, equals to the ratio of AC concentrations in the presence and absence of the analyte (β_n) and, according to equation S26, β_n can be obtained from CD and Abs measurements for the following samples:

1) The origami-AuNRs constructs with aptamer and complementary strand in the absence and presence of analyte ($N_n^{b_0=0}$, $N_n^{b_0}$); Usually *n* is varied with 4-5 values, giving 8-10 samples.

2) The origami-AuNRs constructs in open configuration, i.e., zero hybridization, in the buffer with/without analytes, ($N_{\text{open}}^{b_0=0}$, $N_{\text{open}}^{b_0}$);

To obtain a_0, we prepared 4 constructs with *n* varied from 10 to 13 bp. The corresponding $K_{\text{D1}}(n)$ were calculated using $\Delta G^{\circ}_{theory}$ obtained from the software mfold according to equation S37. We measured the CD and Abs signals for each construct in the presence and absence of a DNA competitor strand with the known K_{D2} (2.28 μM) to gain β_n. The input local concentration (a_0) was obtained as 76±3.8 μM, by fitting β_n with $K_{\text{D1}}(n)$ using equation S38, in the fixed concentration b_0 (12.5 μM), R^2 =0.995 (Figure 2E in the main text).

$$K_{\text{D1}}(n) = e^{\frac{\varepsilon \Delta G^{\circ}_{\text{theory}}(n)}{RT}} \quad (S37)$$

$$\beta_n = \frac{N_n^{*(b_0)}}{N_n^{*(b_0=0)}} = \frac{K_{\text{D1}}(n) + b_0 \frac{K_{\text{D1}}(n)}{K_{\text{D2}}} + 2a_0 - \sqrt{\left(1 + \frac{2b_0}{K_{\text{D2}}} + \frac{b_0^2}{K_{\text{D2}}^2}\right) \cdot K_{\text{D1}}^2(\text{n}) + \left(4a_0 + \frac{4a_0 \text{b}_0}{K_{\text{D2}}}\right) \cdot K_{\text{D1}}(n)}}{2a_0 + K_{\text{D1}}(n) - \sqrt{4a_0 K_{\text{D1}}(n) + K_{\text{D}_1}^2(n)}} \quad (S38)$$

To obtain K_{D2}:

The total input local concentration of aptamer/complementary strand (a_0=76 μM) and the correction coefficient (ε=0.65) remained the same in all experiments as determined in the calibration experiment, as a_0 and ε are intrinsic parameters of the origami-AuNRs construct. The input bulk concentration of analytes (b_0) was a known constant in the experiments. The Gibbs free energy $\Delta G^{\circ}_{\text{theory}}(n)$ was calculated by the mfold software to determine the dissociation constant of the aptamer to the complementary strands ($K_{D1}(n)$) with equation S37. The AC concentration ratio (β_n) in the presence and absence of the analytes equaled to the ratio of the relative normalized CD signal in the presence and absence of the analyte and was calculated form the measured CD and Abs signals. By fitting $K_{D1}(n)$ and β_n with equation S38, the dissociation constant of the aptamer to the analytes (K_{D2}) can be obtained.

Equation used:

Table S5. The fitting equations used.

Name of the fitting	Comments	R^2	Equation	Generated parameter
Fit for ε		0.988	$y = \omega\, ln \frac{(N_{\text{closed}} - x)^2}{x} + c$	ω=3.76±0.37
Fit for a_0		0.998	$y = \frac{12.5 \frac{x}{2.28} + x + 2a_0 - \sqrt{\left(1 + \frac{25}{2.28} + \frac{12.5^2}{2.28^2}\right) x^2 + \left(4a_0 + \frac{50a}{2.28}\right) x}}{2a_0 + x - \sqrt{4a_0 x + x^2}}$	a_0=76.4±3.8
Fit for K_{D2}	ATP conc.[*] Figure 2D main text	0.978	$y = \frac{x \frac{K_{D1}}{K_{D2}} + K_{D1} + 2a_0 - \sqrt{\left(1 + \frac{2x}{K_{D2}} + \frac{x^2}{{K_{D2}}^2}\right) {K_{D1}}^2 + \left(4a_0 + \frac{4a_0 x}{K_{D2}}\right) K_{D1}}}{2a_0 + K_{D1} - \sqrt{4a_0 K_{D1} + {K_{D1}}^2}}$ $K_{D1} = 0.488, a_0 = 76$	K_{D2}=4.54±0.58
Fit for K_{D2}	ATP 5' Figure 2B main text	0.959	$y = \frac{b_0 \frac{x}{K_{D2}} + x + 2a_0 - \sqrt{\left(1 + \frac{2b_0}{K_{D2}} + \frac{{b_0}^2}{{K_{D2}}^2}\right) x^2 + \left(4a_0 + \frac{4a_0 b_0}{K_{D2}}\right) x}}{2a_0 + x - \sqrt{4a_0 x + x^2}}$ $a_0 = 76, b_0 = 10^3 \text{(ATP)}, 10^5 \text{ (glucose)}, 0.17 \text{ (thrombin)}$	K_{D2}=5.71±0.87
	ATP 3' Figure 2C main text	0.128		K_{D2}=117±62
	Glucose 5' Figure 3A main text	0.991		K_{D2}=(5.57±0.44) $\cdot 10^3$

[*] conc. stands for concentration

	Glucose 3'<br>Figure 3B main text	0.916		K_{D2}=(110±20) ·10^3
	Thrombin 5'<br>Figure 3D main text	0.942		K_{D2}=(235±24) ·10^{-3}
	Thrombin 3'<br>Figure 3E main text	0.965		K_{D2}=(46.7±7.3) ·10^{-3}

Parameters used:

Table S6. The $\Delta G^{\circ}_{\mathrm{theory}}$ and the K_{D1} of the template strand and the complementary strands.

The $\Delta G^{\circ}_{\mathrm{theory}}$ (kJ mol^{-1}) and K_{D1} (μM) of the template and the complementary strands							
Hybridization # (bp)	8	9	10	11	12	13	14
$\Delta G^{\circ}_{\mathrm{theory}}$ (kJ/mol)	-38.5	-41.4	-45.2	-55.2	-57.7	-64.0	-72.0
K_{D1} (μM)	39.9	18.5	6.85	0.488	0.252	0.0483	0.00596

Table S7. The K_{D1} of the ATP aptamer and the complementary strands.

The K_{D1} (μM) of ATP aptamer and the complementary strands				
Hybridization # (bp)	9	10	11	12
5’	0.488	0.252	0.0347	0.00829
3’	5.50	0.845	0.0387	0.00308

Table S8. The K_{D1} of the glucose aptamer and the complementary strands.

The K_{D1} (μM) of glucose aptamer and the complementary strands					
Hybridization # (bp)	8	9	10	11	12
5’	3.95	0.608	0.130	0.0179	0.00384
3’	55.5	5.50	0.607	0.252	0.0223

Table S9. The K_{D1} of the thrombin aptamer and the complementary strands.

The K_{D1} (μM) of thrombin aptamer and the complementary strands					
Hybridization # (bp)	7	8	9	10	11
5’	134	18.5	3.95	0.544	0.0672
3’	149	13.3	4.92	0.488	0.0483

To obtain specificity:

The fixed aptamer and complementary strand pair were chosen based on the affinity experiment so that the kinetic trap and side product were avoided. The origami-AuNRs with the fixed aptamer and complementary strand was incubated with different analytes or without any analyte and the normalized CD signal (N) was obtained. The normalized signal of the origami-AuNRs in buffer was set as 100% and the normalized signal of the origami-AuNRs with different analyte treatment was divided by the normalized signal in buffer to gain the percentage. The signal drop percentages in different analytes were compared to qualitatively determine the specificity.

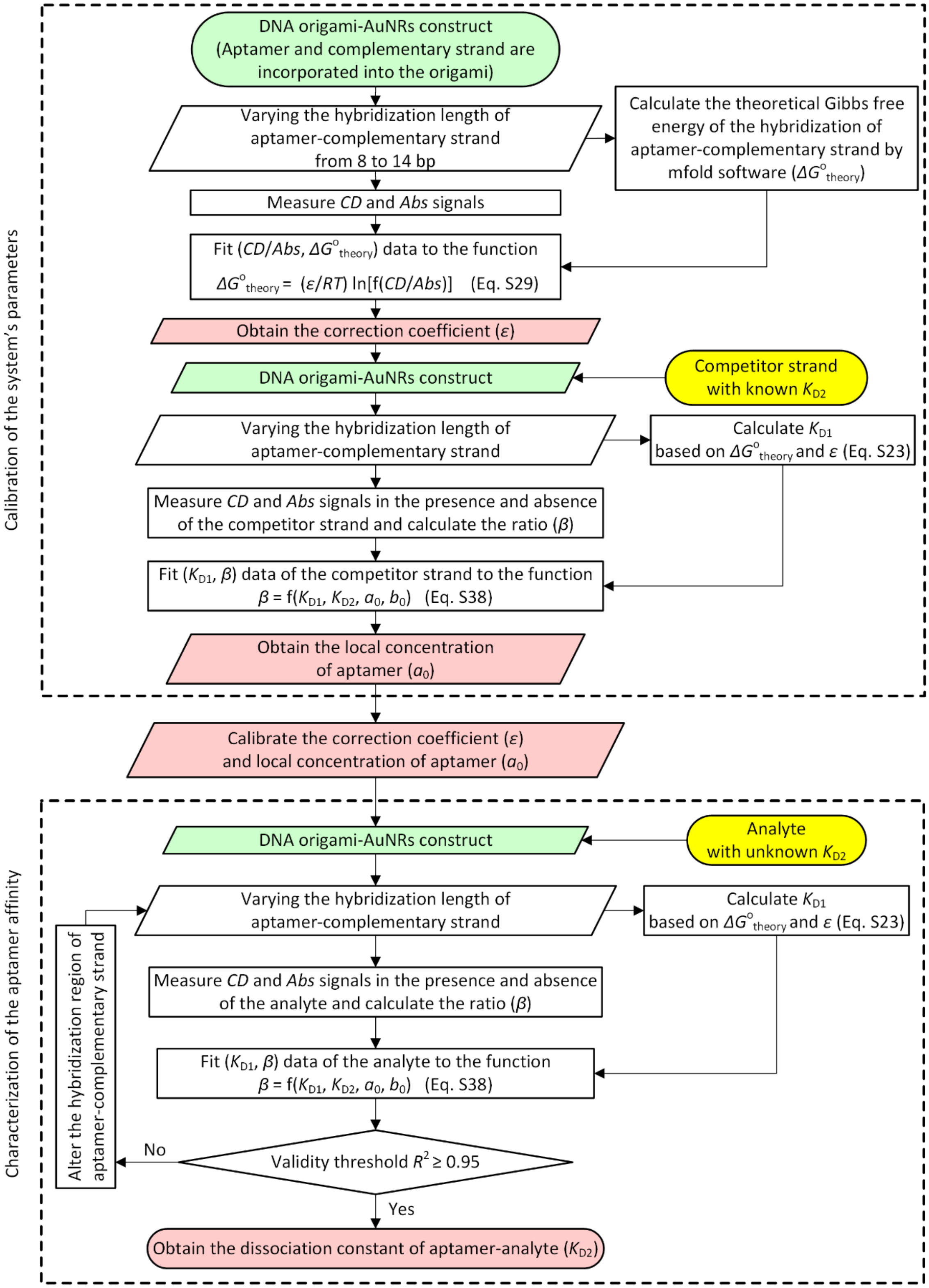

Figure S4. The workflow of the aptamer characterization.

Section 3. Design considerations and benefits of plasmonic chiral probes for the aptamer characterization

Directly measuring the fractions of bound and unbound aptamers relies on finding specific characteristics to differentiate the analyte-aptamer complex and free aptamers, which often change from case to case, and thus dictates different approaches. However, by applying competitive hybridization reactions-based strategy, a unified technique can be applied to various aptamers.

Compared to the traditional model in competitive hybridization reactions-based strategy, where concentration dependency is used with a fixed complementary strand, our approach varies the complementary strands both in hybridization region and lengths at a fixed analyte concentration and is expected to be more generalizable.

Considering the heterogeneous nature of the aptamer domains, typically the whole aptamer sequence can be sepearated into three functional domains (non-essential, essential but non-critical, and critical domains):

i) The non-essential domain contains the sequence that neither interacts with the analyte nor supports structural folding and should be truncated;

ii) The essential but non-critical domain contains the sequence which is not critical for the initial interaction of analyte but essential for analyte binding;

iii) The critical domain contains the sequence that plays important role in the initial interaction of analyte.

Choosing a complementary strand without preliminary knowledge of the aptamer domains suffers from the risk of forming kinetic traps and/or side product, causing unreliable affinity (K_{D}) measurement. Specifically, a complementary strand hybridizing with the non-essential domain of the aptamer, can form the stable analyte-aptamer-complementary strand complex and, consequently, prevent the separation of the aptamer and the complementary strand. In addition, a complementary strand, which blocks the critical domain, can kinetically trap the aptamer at the hybridized state by jeopardizing the induced-fit path, and, consequently, hinders the analyte binding reaction reaching equilibrium when the dissociation rate is slow.

By varying the complementary strands and using the goodness of fitting (R^2), the reliability of the K_{D} measurements can be ensured. Only when the equilibrium is reached and no side product is generated, the ($\beta_n, K_{\mathrm{D1}}(n)$) can fit the model well.

To set a meaningful threshold of R^2 for the judgment of the validity, adjusting β_n values into a proper range is required. If most β_n are close to 1 (CD signals remaining similar to the original levels after the addition of the analyte), the fitting might generate an invalid K_{D2} value with a high R^2 just because the mathmetical calculation of the deviation is small. If most of β_n values are close to 0 (CD signals droping to 0 after the addition of the analyte), the fitting might generate a valid K_{D2} value with a low R^2 because the system erros become large when the signals are small. Therefore, as $\beta = f(a_0, b_0, K_{\mathrm{D1}}, K_{\mathrm{D2}})$, we typically used the fixed concentration of analyte (b_0) at approximately 10-100 times of the K_{D2} value so the values of β_n fall into the range of 0.2-0.99. The hybridization length (n) of the aptamer and complementary strand falls into the 7-12 bp range to ensure the initial signal is large enough while the two state model of DNA hybridization and separation is still valid.

The complementary strands used in this work hybridize either to 5' or 3' end of aptamers as the valid K_{D2} values have already been obtained from one end. However, in principle, the

complementary strands can map over the whole aptamer when the essential but non-critical domain is not contained by the end sequence.

The appraoch is expected to be generalizable because in principle, all aptamers should contain an essential but non-critical sequence. The ‘aptamer’ that only contains non-essential sequence is not an aptamer. There is, however, a possibility that the aptamer only contains the critical domain so introducing any complementary strands will block the induced-fit path and create a kinetic trap. To solve the issue, after obtaining the information of the aptamer domain, a fixed short complementary sequence (8-9 bp), which allows fast dissociation, together with the concentration dependency fitting can be used as we have demonstrated in the Figure 3D.

In some specific cases, where the aptamers have significant amount of base pairs (e.g. RNA aptamer against ATP, RNA aptamer against HIV-1 Tat, DNA aptamer against cocaine etc.), the hetero-hybridization has to compete against the self-hybridization to form the hybrid. To keep the Gibbs free energy of the hybridization reaction similar to the general cases, a more stable hybridization between the aptamer and the complementary strand is required for these special cases as the Gibbs free energy of the product has to be low to compensate for the low Gibbs free energy of the reactants. Consequently, the slow dissociation rate problem arises here. In this case, the complementary strand allowing the induced-fit path has to be guaranteed. In rare cases, the complementary strand may act as a partial split aptamer as it contains the same or similar sequence of the parent aptamer and thus fail to be competed off by the analyte binding. In this scenario, the competitive hybridization reaction-based model should be replaced by the sandwich model of the split aptamer and analyte.

The plasmonic chiral probes provide the feasibility of the approach of varying complementary strands to the aptamer due to the high signal-to-noise ratio, allowing the system to differentiate the change of a single base pair (Figure 2B). The traditional fluorescence-based probes rely on a stable binding between aptamer and its complementary strand to reduce the background noise, which may hinder the system from reaching equilibrium. The common approach to solving this kinetical trap problem is the addition of extra nucleotides to the aptamer sequence to provide a longer hybridization length for the complementary strand while maintaining the critical domain accessible by the analyte. This, however, may cause the side product of the analyte-aptamer-complementary strand complex and is only possible for the hybridization starting from the ends of the aptamer.

Section 4. Supporting data

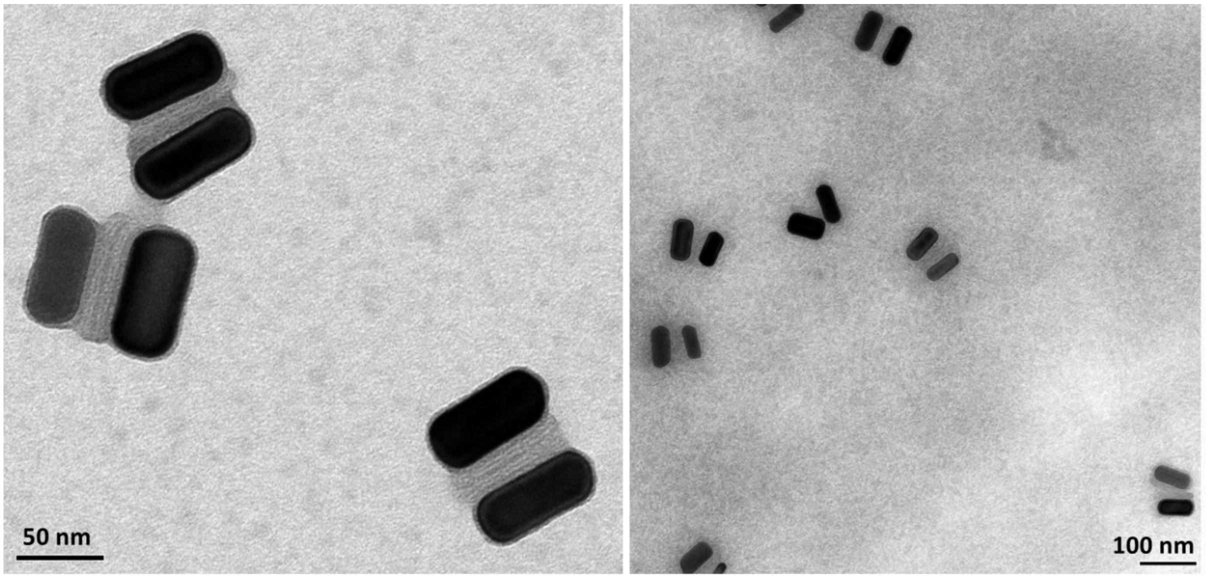

Figure S5. TEM images of the origami-AuNRs.

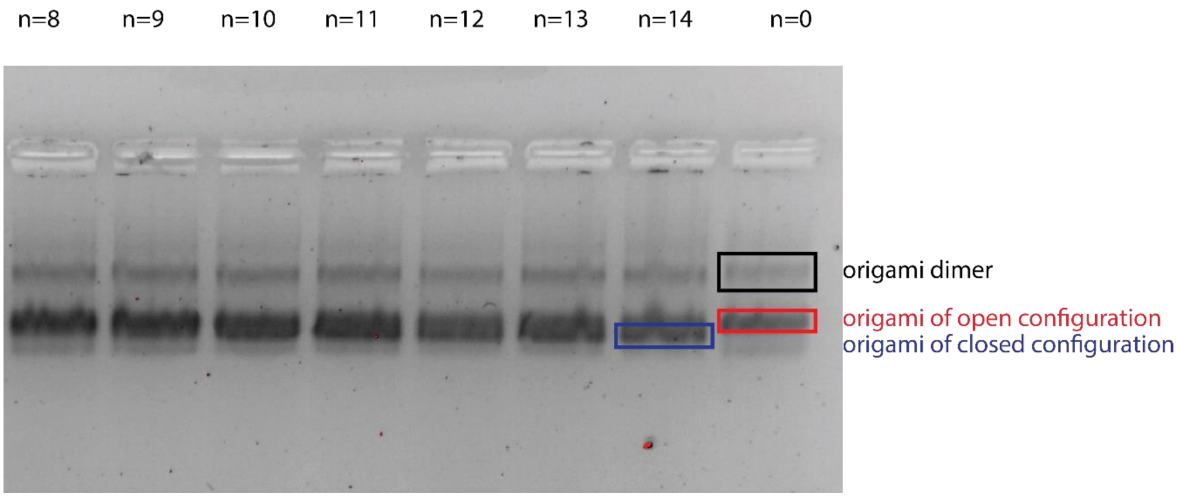

Figure S6. Gel electrophoresis of origami with DNA template strand (A*) and its complementary strands (C) of various lengths. The gel was run in 2% agarose gel for 4 h at 100 V and then stained by Sybrgold for 40 min. The origami shifts from open (higher band) to close (lower band) when the hybridization lengths increase.

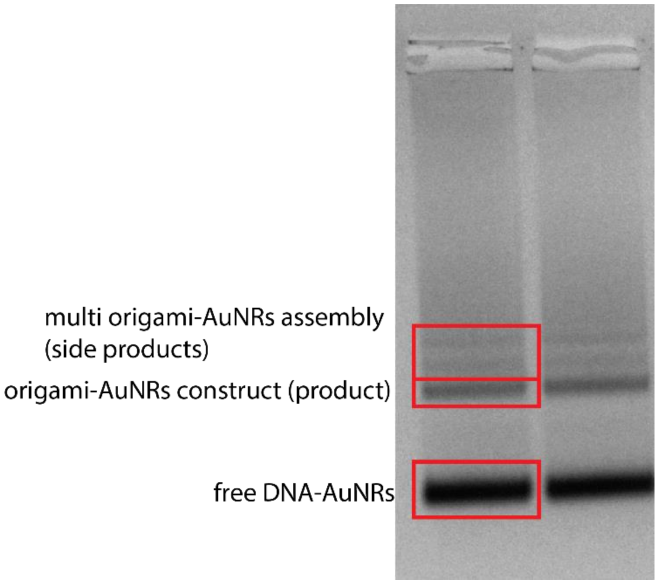

Figure S7. The origami-AuNRs samples were purified by gel electrophoresis with a 0.7% agarose gel at 80 V for 3 h.

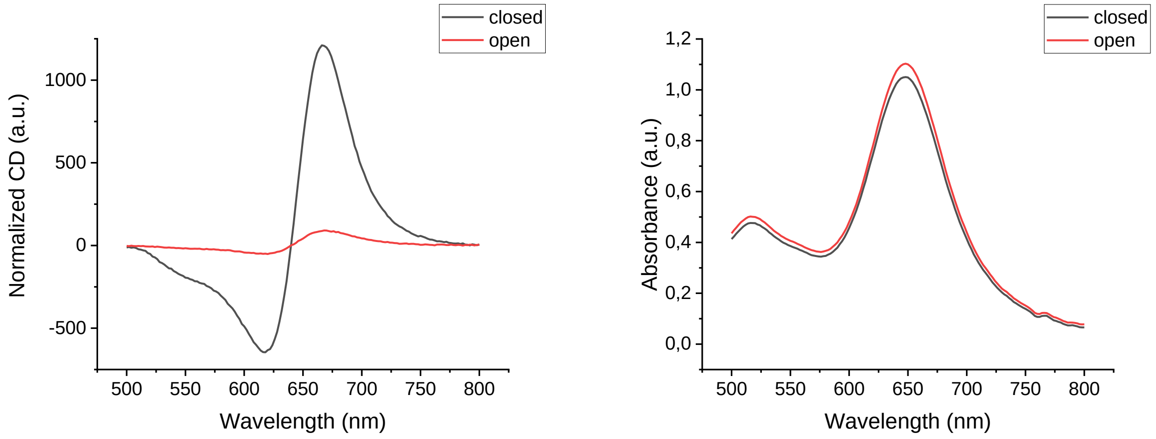

Figure S8. The normalized CD (left) and the Abs (right) spectra of the origami-AuNRs chiral probes in open and closed states.

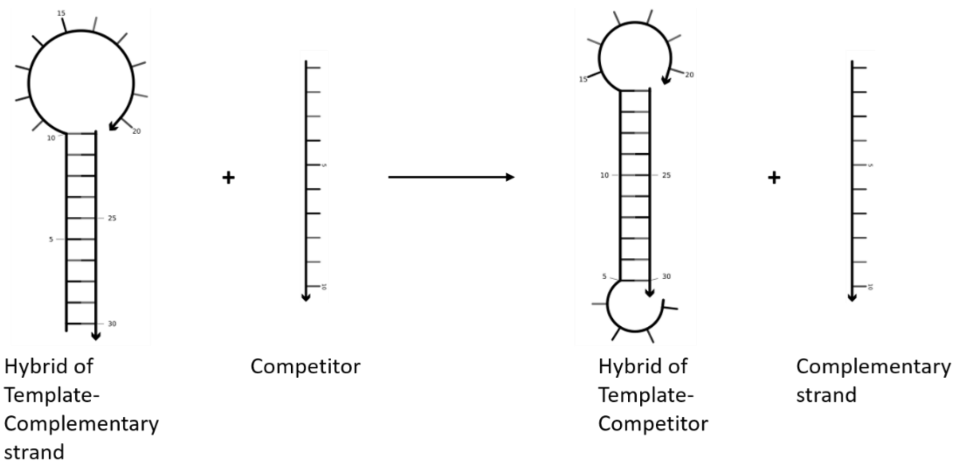

Figure S9. The strand displacement reaction of the competitor strand for complementary strand, using *n*=10 bp as an example. The structure was generated and adapted from NUPACK.[11]

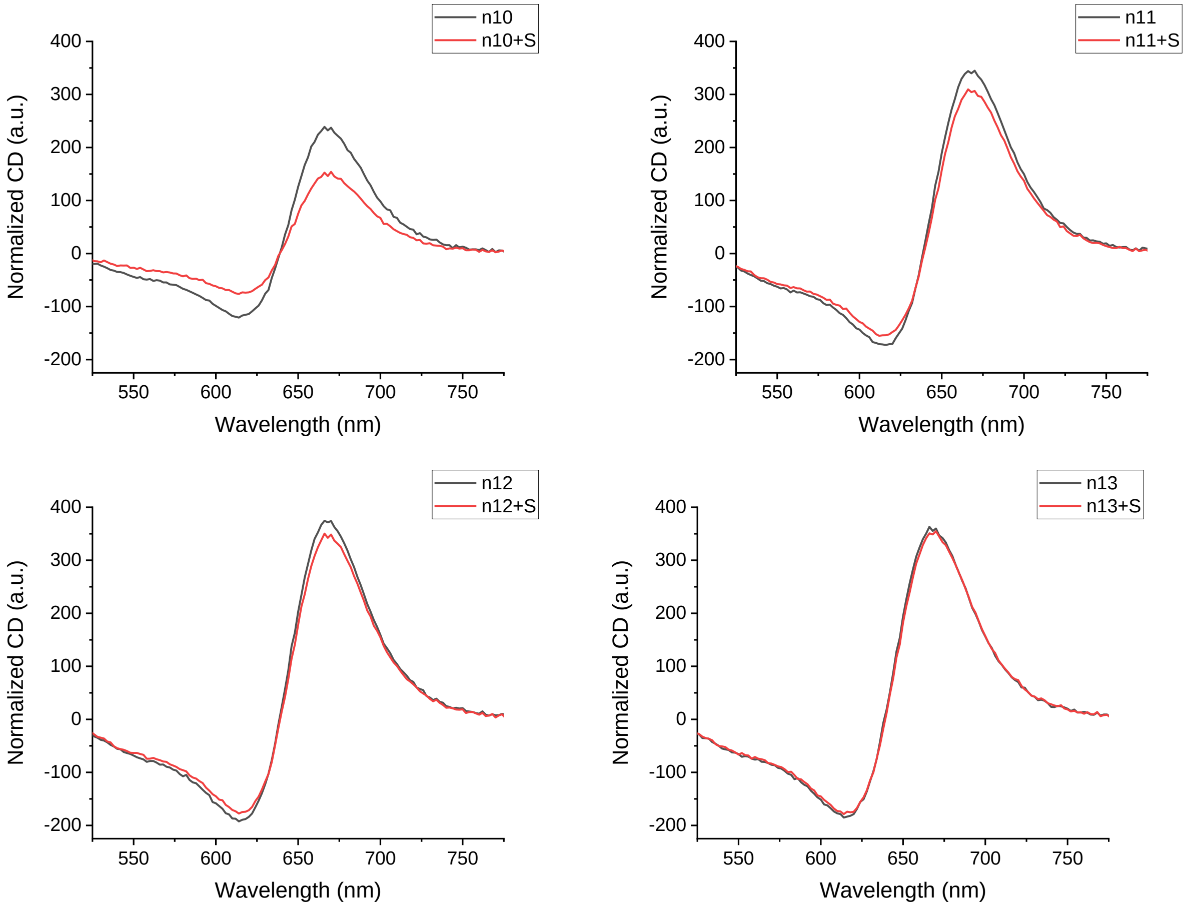

Figure S10. The normalized CD spectra of the origami-AuNRs with various hybridization lengths *n*={10,11,12,13} of the template strand-complementary strands incubated with and without the competitor strand (S).

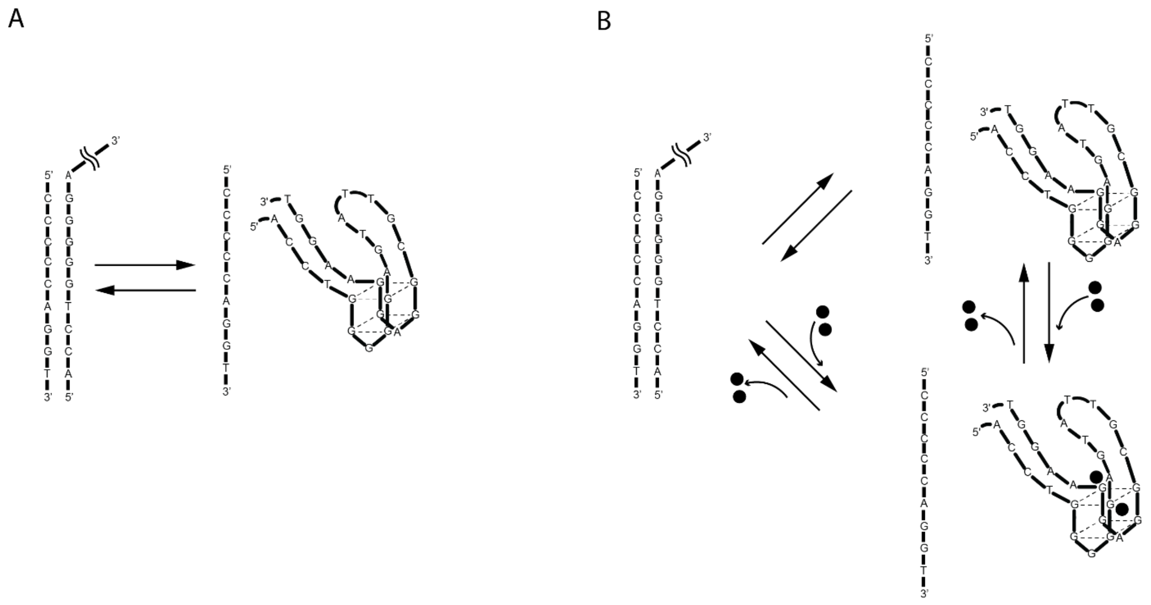

Figure S11. The hybridization of the ATP aptamer and its complementary strand (using *n*=9 bp, hybridizing from 5' end of the aptamer as an example) in the absence (**A**) and presence (**B**) of the ATP.

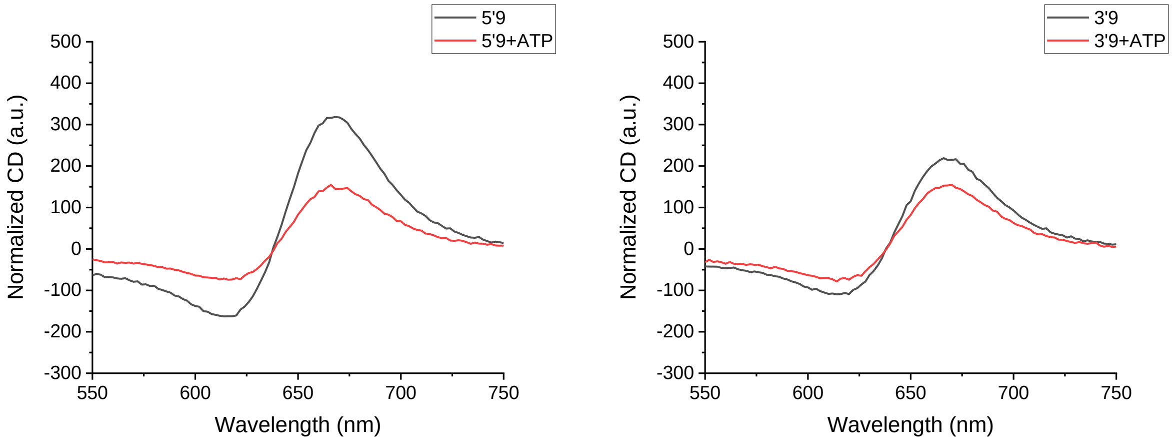

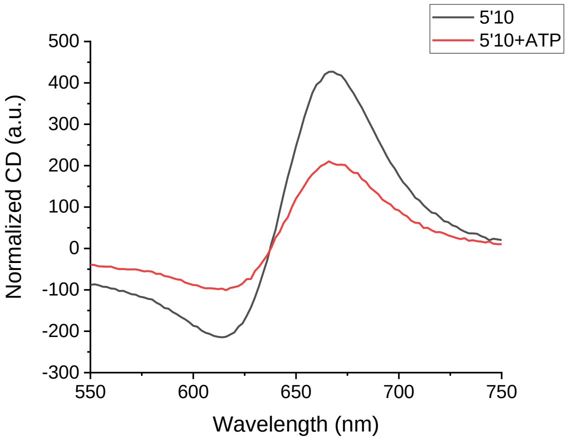
5'10
5'10+ATP
Normalized CD (a.u.)
500
400
300
200
100
0
-100
-200
-300
550
600
650
700
750
Wavelength (nm)

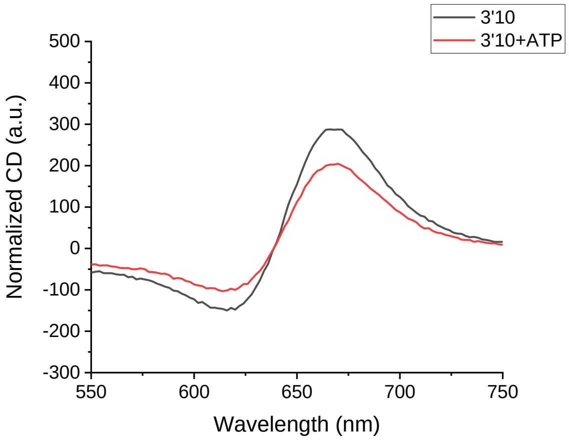
3'10
3'10+ATP
Normalized CD (a.u.)
500
400
300
200
100
0
-100
-200
-300
550
600
650
700
750
Wavelength (nm)

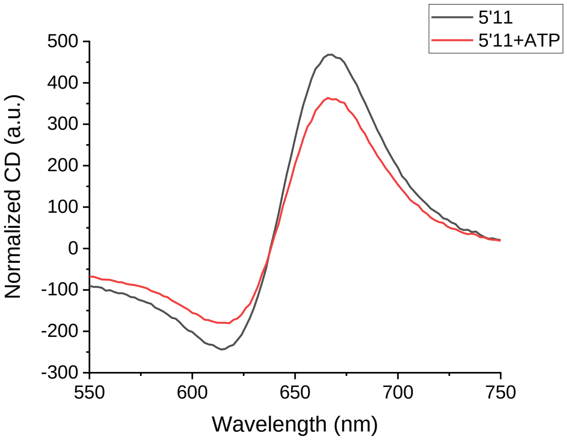
5'11
5'11+ATP
Normalized CD (a.u.)
500
400
300
200
100
0
-100
-200
-300
550
600
650
700
750
Wavelength (nm)

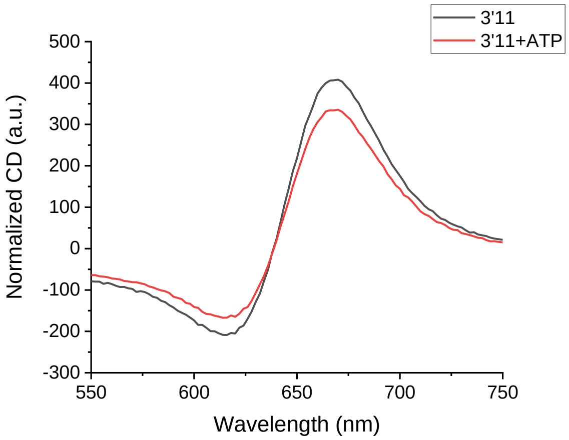
3'11
3'11+ATP
Normalized CD (a.u.)
500
400
300
200
100
0
-100
-200
-300
550
600
650
700
750
Wavelength (nm)

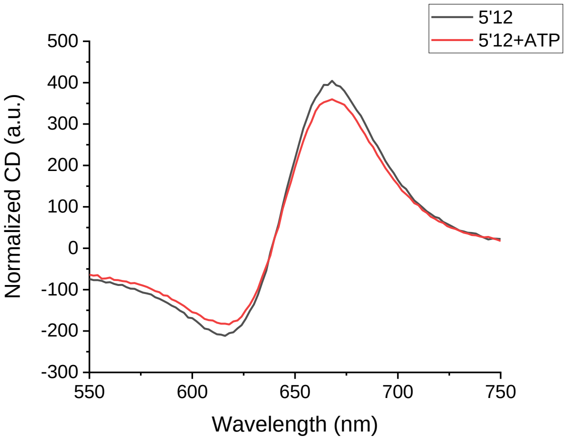
5'12
5'12+ATP
Normalized CD (a.u.)
500
400
300
200
100
0
-100
-200
-300
550
600
650
700
750
Wavelength (nm)

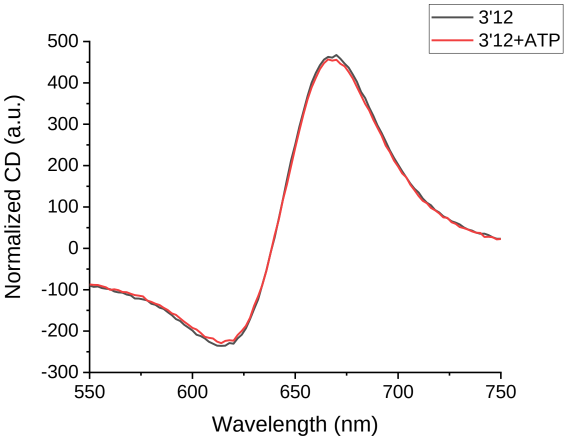
3'12
3'12+ATP
Normalized CD (a.u.)
500
400
300
200
100
0
-100
-200
-300
550
600
650
700
750
Wavelength (nm)

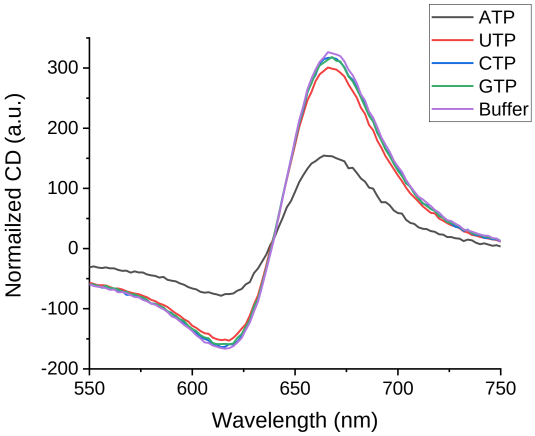

Figure S12. The normalized CD spectra of the origami-AuNRs with various hybridization lengths of the ATP aptamer-complementary strands incubated with and without ATP (1mM). The origami-AuNRs with n=9 bp hybridization length from aptamer 5' end incubated with ATP, UTP, CTP, GTP (1 mM).

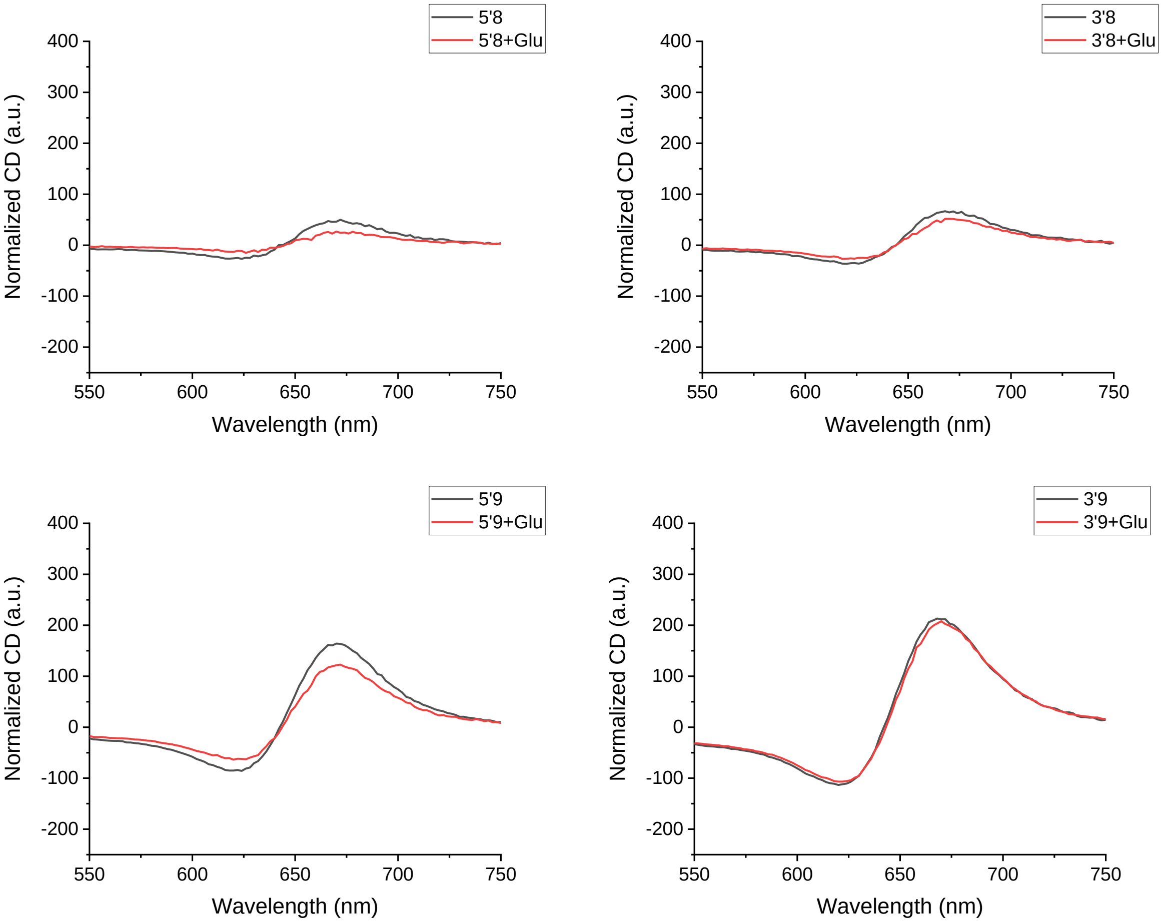

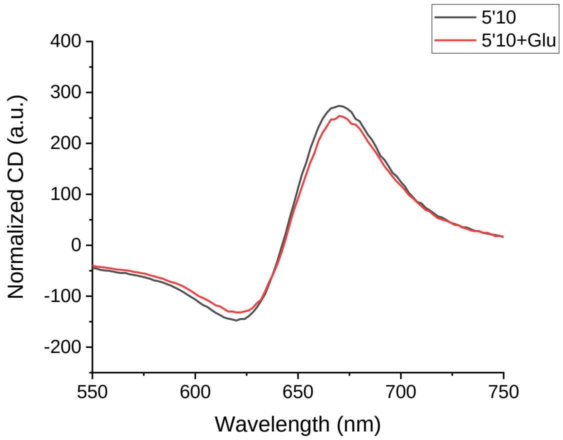
5'10
5'10+Glu
Normalized CD (a.u.)
Wavelength (nm)
400
300
200
100
0
-100
-200
550
600
650
700
750

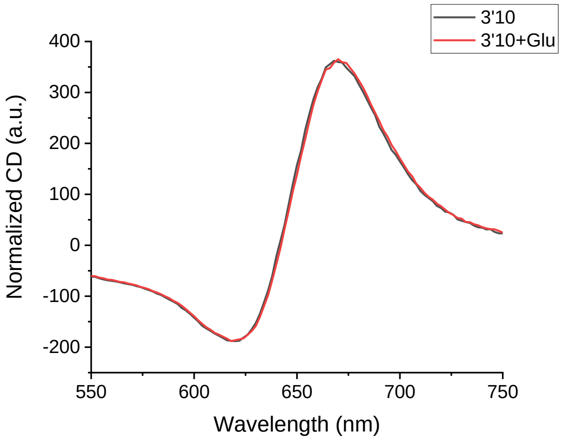
3'10
3'10+Glu
Normalized CD (a.u.)
Wavelength (nm)
400
300
200
100
0
-100
-200
550
600
650
700
750

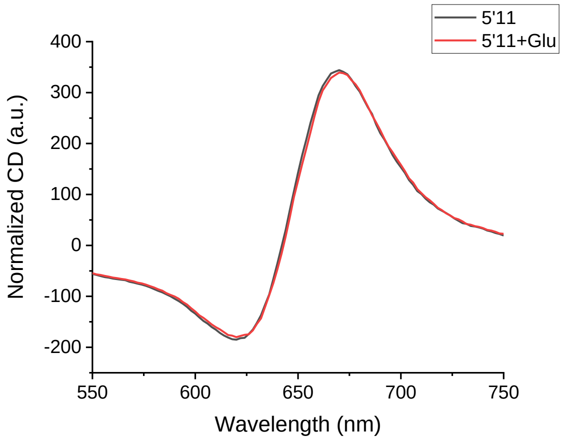
5'11
5'11+Glu
Normalized CD (a.u.)
Wavelength (nm)
400
300
200
100
0
-100
-200
550
600
650
700
750

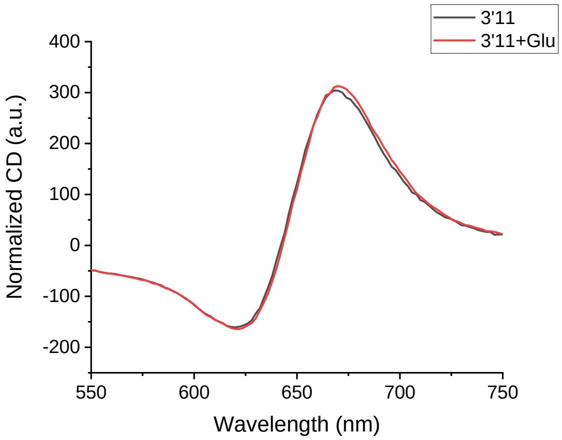
3'11
3'11+Glu
Normalized CD (a.u.)
Wavelength (nm)
400
300
200
100
0
-100
-200
550
600
650
700
750

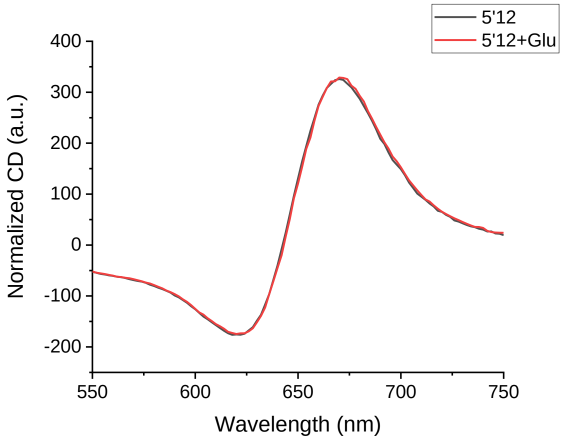
5'12
5'12+Glu
Normalized CD (a.u.)
Wavelength (nm)
400
300
200
100
0
-100
-200
550
600
650
700
750

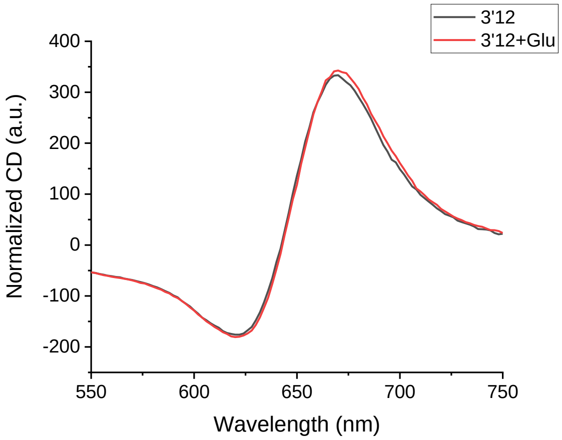
3'12
3'12+Glu
Normalized CD (a.u.)
Wavelength (nm)
400
300
200
100
0
-100
-200
550
600
650
700
750

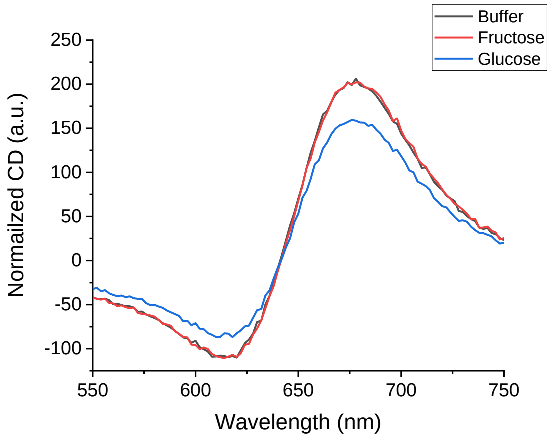

Figure S13. The normalized CD spectra of the origami-AuNRs with various hybridization length of the glucose aptamer strand-complementary strands incubated with and without glucose (100 mM). The origami-AuNRs with 10 bp hybridization length from aptamer 5' end incubated with fructose and glucose (100 mM).

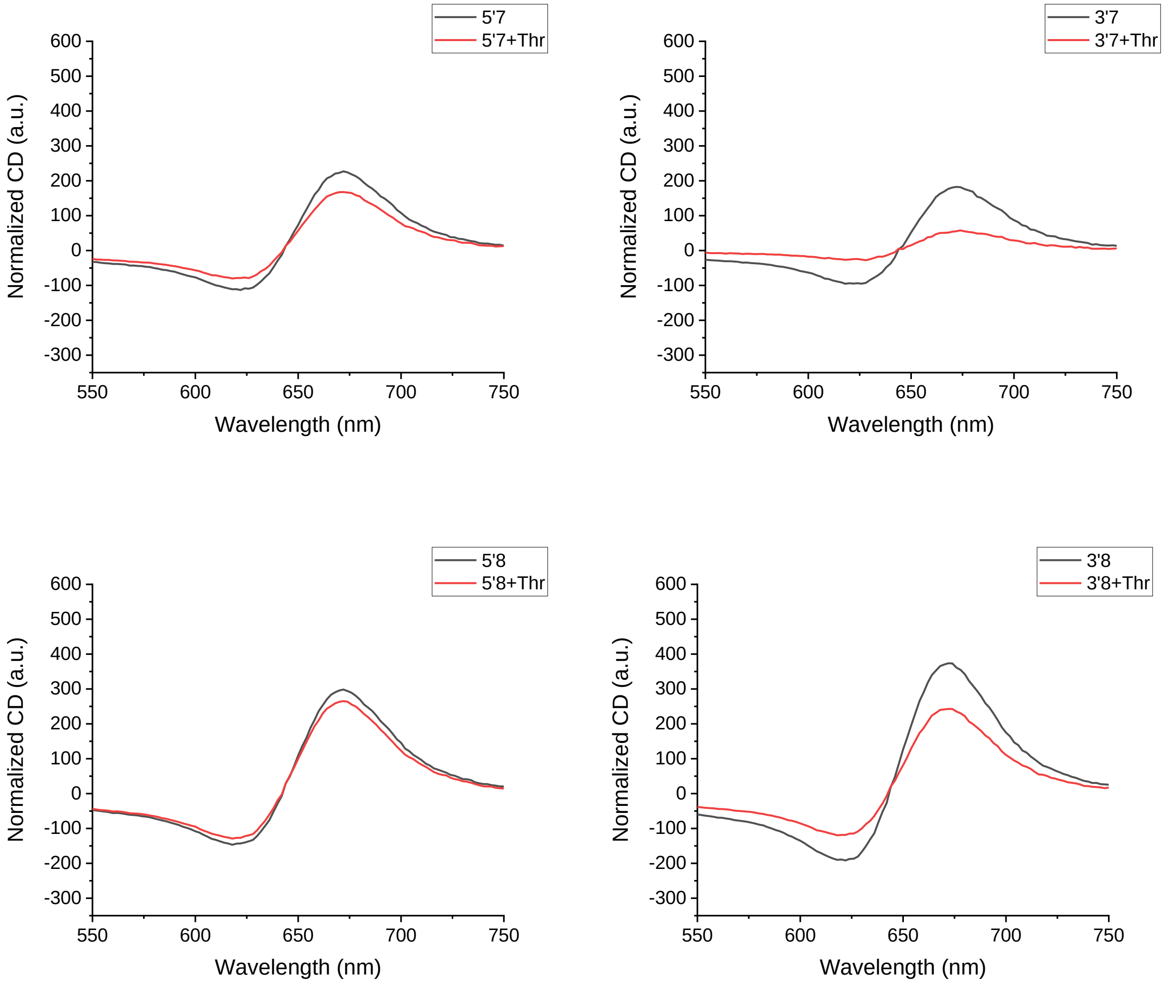

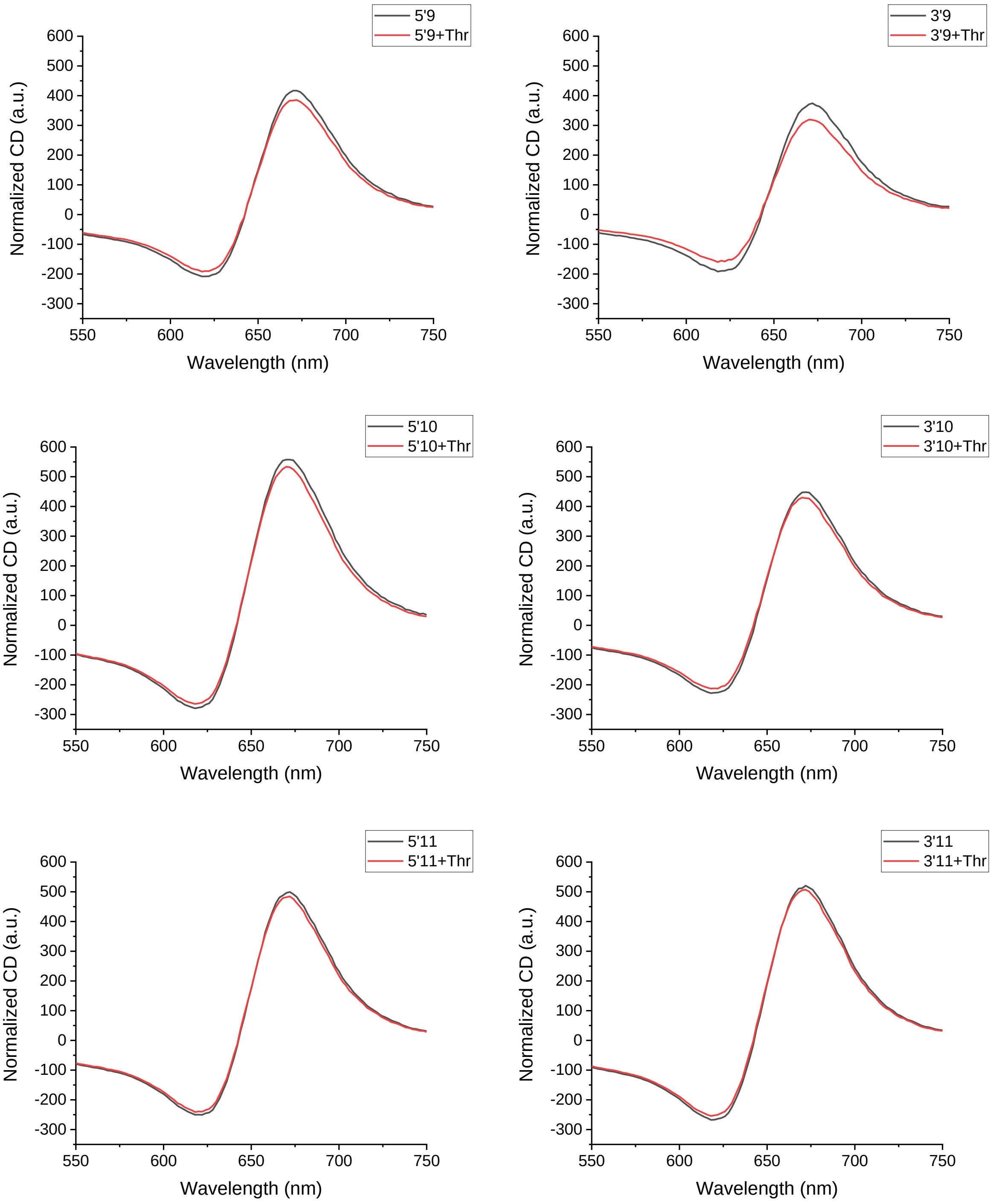
5'9
5'9+Thr
3'9
3'9+Thr
5'10
5'10+Thr
3'10
3'10+Thr
5'11
5'11+Thr
3'11
3'11+Thr
Normalized CD (a.u.)
Wavelength (nm)
600
500
400
300
200
100
0
-100
-200
-300
550
600
650
700
750

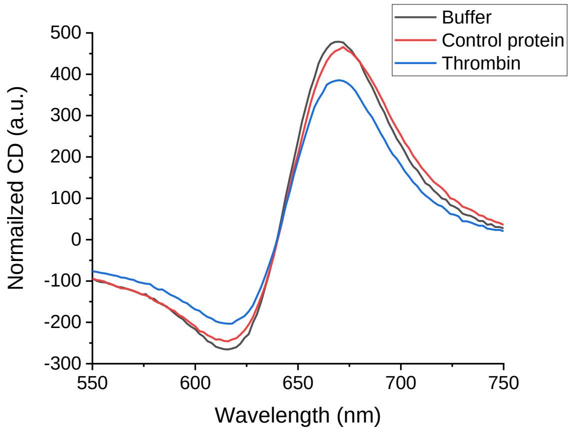

Figure S14. The normalized CD spectra of the origami-AuNRs with various hybridization length of the thrombin aptamer strand-complementary strands incubated with and without thrombin (170 nM). The origami-AuNRs with 8 bp hybridization length from aptamer 3' end incubated with control protein (2 µM) and thrombin (170 nM).

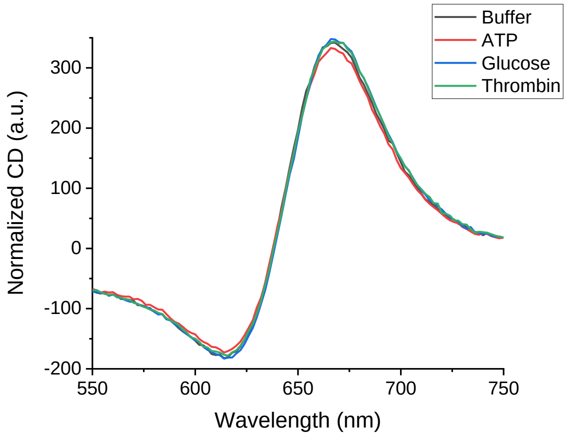

Figure S15. The normalized CD spectra of the origami-AuNRs with the lock of the template sequence and its complementary strand (10 bp) in different analytes (1 mM ATP, 100 mM Glucose, 170 nM thrombin). As the lock contained no aptamer sequence, the probe response in the maximum concentration of the analyte is minimal.

References

(1) Huizenga, D. E.; Szostak, J. W. A DNA Aptamer That Binds Adenosine and ATP. *Biochemistry* **1995**, *34* (2), 656–665. https://doi.org/10.1021/bi00002a033.

(2) Nakatsuka, N.; Yang, K.-A.; Abendroth, J. M.; Cheung, K. M.; Xu, X.; Yang, H.; Zhao, C.; Zhu, B.; Rim, Y. S.; Yang, Y.; Weiss, P. S.; Stojanović, M. N.; Andrews, A. M. Aptamer–Field-Effect

Transistors Overcome Debye Length Limitations for Small-Molecule Sensing. *Science* **2018**, *362* (6412), 319–324. https://doi.org/10.1126/science.aao6750.

(3) Bock, L. C.; Griffin, L. C.; Latham, J. A.; Vermaas, E. H.; Toole, J. J. Selection of Single-Stranded DNA Molecules That Bind and Inhibit Human Thrombin. *Nature* **1992**, *355* (6360), 564–566. https://doi.org/10.1038/355564a0.

(4) Douglas, S. M.; Marblestone, A. H.; Teerapittayanon, S.; Vazquez, A.; Church, G. M.; Shih, W. M. Rapid Prototyping of 3D DNA-Origami Shapes with CaDNAno. *Nucleic Acids Research* **2009**, *37* (15), 5001–5006. https://doi.org/10.1093/nar/gkp436.

(5) Ye, X.; Jin, L.; Caglayan, H.; Chen, J.; Xing, G.; Zheng, C.; Doan-Nguyen, V.; Kang, Y.; Engheta, N.; Kagan, C. R.; Murray, C. B. Improved Size-Tunable Synthesis of Monodisperse Gold Nanorods through the Use of Aromatic Additives. *ACS Nano* **2012**, *6* (3), 2804–2817. https://doi.org/10.1021/nn300315j.

(6) Huang, Y.; Nguyen, M.-K.; Kuzyk, A. Assembly of Gold Nanorods into Chiral Plasmonic Metamolecules Using DNA Origami Templates. *JoVE (Journal of Visualized Experiments)* **2019**, No. 145, e59280. https://doi.org/10.3791/59280.

(7) Zhang, X.; Servos, M. R.; Liu, J. Instantaneous and Quantitative Functionalization of Gold Nanoparticles with Thiolated DNA Using a pH-Assisted and Surfactant-Free Route. *J. Am. Chem. Soc.* **2012**, *134* (17), 7266–7269. https://doi.org/10.1021/ja3014055.

(8) Shi, D.; Song, C.; Jiang, Q.; Wang, Z.-G.; Ding, B. A Facile and Efficient Method to Modify Gold Nanorods with Thiolated DNA at a Low PH Value. *Chem. Commun.* **2013**, *49* (25), 2533–2535. https://doi.org/10.1039/C3CC39093D.

(9) Zuker, M. Mfold Web Server for Nucleic Acid Folding and Hybridization Prediction. *Nucleic Acids Research* **2003**, *31* (13), 3406–3415. https://doi.org/10.1093/nar/gkg595.

(10) Idili, A.; Ricci, F.; Vallée-Bélisle, A. Determining the Folding and Binding Free Energy of DNA-Based Nanodevices and Nanoswitches Using Urea Titration Curves. *Nucleic Acids Research* **2017**, *45* (13), 7571–7580. https://doi.org/10.1093/nar/gkx498.

(11) Zadeh, J. N.; Steenberg, C. D.; Bois, J. S.; Wolfe, B. R.; Pierce, M. B.; Khan, A. R.; Dirks, R. M.; Pierce, N. A. NUPACK: Analysis and Design of Nucleic Acid Systems. *Journal of Computational Chemistry* **2011**, *32* (1), 170–173. https://doi.org/10.1002/jcc.21596.
